# Supplementary material for: Designing a comprehensive behaviour change intervention to promote and monitor exclusive use of liquefied petroleum gas stoves for the Household Air Pollution Intervention Network (HAPIN) trial
Source: BMJ Open. 2020 Sep 29;10(9):e037761. doi: 10.1136/bmjopen-2020-037761 (PMC7526279; doi:10.1136/bmjopen-2020-037761)
Supplement: Author's manuscript [file bmjopen-2020-037761.draft_revisions.pdf]

BMJ Open is committed to open peer review. As part of this commitment we make the peer review history of every article we publish publicly available.

When an article is published we post the peer reviewers' comments and the authors' responses online. We also post the versions of the paper that were used during peer review. These are the versions that the peer review comments apply to.

The versions of the paper that follow are the versions that were submitted during the peer review process. They are not the versions of record or the final published versions. They should not be cited or distributed as the published version of this manuscript.

BMJ Open is an open access journal and the full, final, typeset and author-corrected version of record of the manuscript is available on our site with no access controls, subscription charges or pay-per-view fees (http://bmjopen.bmj.com).

If you have any questions on BMJ Open's open peer review process please email info.bmjopen@bmj.com

BMJ Open

Designing a comprehensive behavior change framework to promote and monitor exclusive use of liquefied petroleum gas stoves for the Household Air Pollution Intervention Network (HAPIN) trial

Journal:	*BMJ Open*
Manuscript ID	bmjopen-2020-037761
Article Type:	Original research
Date Submitted by the Author:	17-Feb-2020
Complete List of Authors:	Williams, Kendra; Johns Hopkins School of Medicine, Division of Pulmonary and Critical Care Medicine; Johns Hopkins School of Medicine, Center for Global Non-Communicable Disease Research and Training<br>Thompson, Lisa; Emory University, Nell Hodgson Woodruff School of Nursing<br>Sakas, Zoe; London School of Hygiene and Tropical Medicine Faculty of Infectious and Tropical Diseases, Disease Control<br>Hengstermann, Mayari; Universidad del Valle de Guatemala Centro de Estudios en Salud<br>Quinn, Ashlinn; National Institutes of Health, Fogarty International Center<br>Díaz Artiga, Anaité; Universidad del Valle de Guatemala, Centro de Estudios en Salud<br>Thangavel, Gurusamy; Sri Ramachandra Medical College and Research Institute, Department of Environmental Health Engineering<br>Puzzolo, Elisa; University of Liverpool, Public Health and Policy<br>Rosa, Ghislaine; London School of Hygiene and Tropical Medicine Faculty of Infectious and Tropical Diseases, Department of Disease Control<br>Balakrishnan, Kalpana; Sri Ramachandra Medical College and Research Institute, Department of Environmental Health Engineering<br>Peele, Jennifer; Colorado School of Public Health, Environmental & Radiological Health Sciences<br>Checkley, William; Johns Hopkins School of Medicine, Division of Pulmonary and Critical Care Medicine; Johns Hopkins School of Medicine, Center for Global Non-Communicable Disease Research and Training<br>Clasen, Thomas; Emory University, Rollins School of Public Health<br>Miranda, J. Jaime; Universidad Peruana Cayetano Heredia, CRONICAS Center of Excellence in Chronic Diseases<br>Rosenthal, Joshua; National Institutes of Health, Fogarty International Center<br>Harvey, Steven; Johns Hopkins University Bloomberg School of Public Health, International Health, Social and Behavioral Interventions
Keywords:	PUBLIC HEALTH, QUALITATIVE RESEARCH, Community child health < PAEDIATRICS

SCHOLARONE™
Manuscripts

I, the Submitting Author has the right to grant and does grant on behalf of all authors of the Work (as defined in the below author licence), an exclusive licence and/or a non-exclusive licence for contributions from authors who are: i) UK Crown employees; ii) where BMJ has agreed a CC-BY licence shall apply, and/or iii) in accordance with the terms applicable for US Federal Government officers or employees acting as part of their official duties; on a worldwide, perpetual, irrevocable, royalty-free basis to BMJ Publishing Group Ltd ("BMJ") its licensees and where the relevant Journal is co-owned by BMJ to the co-owners of the Journal, to publish the Work in this journal and any other BMJ products and to exploit all rights, as set out in our licence.

The Submitting Author accepts and understands that any supply made under these terms is made by BMJ to the Submitting Author unless you are acting as an employee on behalf of your employer or a postgraduate student of an affiliated institution which is paying any applicable article publishing charge ("APC") for Open Access articles. Where the Submitting Author wishes to make the Work available on an Open Access basis (and intends to pay the relevant APC), the terms of reuse of such Open Access shall be governed by a Creative Commons licence – details of these licences and which Creative Commons licence will apply to this Work are set out in our licence referred to above.

Other than as permitted in any relevant BMJ Author's Self Archiving Policies, I confirm this Work has not been accepted for publication elsewhere, is not being considered for publication elsewhere and does not duplicate material already published. I confirm all authors consent to publication of this Work and authorise the granting of this licence.

For peer review only

Designing a comprehensive behavior change framework to promote and monitor exclusive use of liquefied petroleum gas stoves for the Household Air Pollution Intervention Network (HAPIN) trial

Kendra N. WILLIAMS[1,2], Lisa M. THOMPSON[3] (co-first), Zoe SAKAS[4], Mayari HENGSTERMANN[5], Ashlinn QUINN[6], Anaité DIAZ ARTIGA[5], Gurusamy THANGAVEL[7], Elisa PUZZOLO[8], Ghislaine ROSA[4], Kalpana BALAKRISHNAN[7], Jennifer PEEL[9], William CHECKLEY[1,2], Thomas F. CLASEN[10], J. Jaime MIRANDA[11], Joshua P. ROSENTHAL[6], Steven A. HARVEY[11], and the HAPIN Investigators[†]

Order and affiliations of authors

1. Division of Pulmonary and Critical Care Medicine, School of Medicine, Johns Hopkins University
2. Center for Global Non-Communicable Disease Research and Training, School of Medicine, Johns Hopkins University, Baltimore, MD 21287
3. Nell Hodgson Woodruff School of Nursing, Emory University, Atlanta, GA 30322, ORCID ID: 0000-0002-8001-2057
4. Department of Disease Control, Faculty of Infectious and Tropical Diseases, London School of Hygiene & Tropical Medicine, London, UK
5. Centro de Estudios en Salud, Universidad del Valle de Guatemala, Guatemala City, Guatemala
6. Fogarty International Center, National Institutes of Health, Bethesda MD 20892*
7. Center for Advanced Research on Air Quality, Climate and Health, Department of Environmental Health Engineering, Sri Ramachandra Institute for Higher Education and Research, India
8. Department of Public Health and Policy, University of Liverpool, Liverpool, UK
9. Department of Environmental & Radiological Health Sciences, Colorado School of Public Health, Colorado State University, Fort Collins, CO 80523
10. Rollins School of Public Health, Emory University, Atlanta, GA 30322
11. CRONICAS Center of Excellence in Chronic Diseases, Universidad Peruana Cayetano Heredia, Lima, Peru
12. Department of International Health, Social and Behavioral Interventions Program, Johns Hopkins Bloomberg School of Public Health, Baltimore, MD 21205

[†] Household Air Pollution Intervention Network (HAPIN) trial Investigators:

Vigneswari Aravindalochanan, Kalpana Balakrishnan, Dana Boyd Barr, Vanessa Burrowes, Alejandra Bussalleu, Devan Campbell, Eduardo Canuz, Adly Castañaza, Howard Chang, William Checkley, Yunyun Chen, Marilú Chiang, Maggie L. Clark, Thomas Clasen, Rachel Craik, Mary Crocker, Victor Davila-Roman, Lisa de las Fuentes, Oscar De León, Anaité Diaz-Artiga, Ephrem Dusabimana, Lisa Elon, Juan Gabriel Espinoza, Irma Sayury Pineda Fuentes,

For peer review only - http://bmjopen.bmj.com/site/about/guidelines.xhtml

Sarada Garg, Dina Goodman, Savannah Gupton, Meghan Hardison, Stella Hartinger, Steven A. Harvey, Mayari Hengstermann, Phabiola M. Herrera, Shakir Hossen, Penelope Howards, Lindsay Jaacks, Shirin Jabbarzadeh, MichaelA.Johnson, Abigail Jones, Katherine Kearns, Miles Kirby, Jacob Kremer, Margaret A. Laws, Pattie Lenzen, Jiawen Liao, Amy E. Lovvorn, Fiona Majorin, Eric McCollum, John McCracken, Julia N. McPeek, Rachel Meyers, J. Jaime Miranda, Erick Mollinedo, Lawrence Moulton, Krishnendu Mukhopadhyay, Luke Naeher, Abidan Nambajimana, Durairaj Natesan, Florien Ndagijimana, Azhar Nizam, Jean de Dieu Ntivuguruzwa, Aris Papageorghiou, Jennifer Peel, Ricardo Piedrahita, Ajay Pillarisetti, Naveen Puttaswamy, Elisa Puzzolo, Ashlinn K. Quinn, Sarah Rajkumar, Usha Ramakrishnan, Rengaraj Ramasami, Davis Reardon, Ghislaine Rosa, Joshua P. Rosenthal, Barry Ryan, Sudhakar Saidam, Zoe Sakas, Sankar Sambandam, Jeremy A. Sarnat, Suzanne Simkovich, Sheela S. Sinharoy, Kirk R. Smith, Kyle Steenland, Damien Swearing, Gurusamy Thangavel, Lisa M. Thompson, Ashley Toenjes, Lindsay Underhill, Jean Damascene Uwizeyimana, Viviane Valdes, Kayla Valentine, Amit Verma, Lance Waller, Megan Warnock, Kendra N. Williams, Wenlu Ye, Bonnie Young

For peer review only

STRUCTURED ABSTRACT:

Introduction: Increasing use of cleaner fuels, such as liquefied petroleum gas (LPG), and dis-adoption of solid fuels is key to reducing household air pollution and realizing potential health improvements in low-income countries. However, achieving exclusive LPG use in households unaccustomed to LPG requires substantial behavior change. We conducted theory-grounded formative research to identify contextual factors influencing cooking fuel choice to guide the development of behavioral strategies for the Household Air Pollution Intervention Network (HAPIN) trial. The HAPIN trial aims to assess the impact of exclusive LPG use on air pollution exposure and health of pregnant women, older adult women, and infants under one year of age in Guatemala, India, Peru, and Rwanda.

Methods: Using the Capability, Opportunity, Motivation–Behavior (COM-B) framework and Behavior Change Wheel (BCW) to guide formative research, we conducted in-depth interviews, focus group discussions, observations, key informant interviews, and pilot studies to identify key influencers of cooking behaviors in the four countries. We used these findings to develop behavioral strategies likely to achieve exclusive LPG use in the HAPIN trial.

Results: We identified nine potential influencers of exclusive LPG use, including perceived disadvantages of solid fuels, family preferences, cookware, traditional foods, non-food related cooking, heating needs, LPG awareness, safety, and cost and availability of fuel. Mapping formative findings onto the theoretical frameworks, behavioral strategies for achieving exclusive LPG use in each research site included free fuel deliveries, locally-acceptable stoves and equipment, hands-on training, and printed materials and videos emphasizing relevant messages. In the HAPIN trial we will monitor and reinforce exclusive LPG use through temperature data loggers, LPG delivery tracking, in-home observations, and behavioral reinforcement visits.

Conclusion: Our formative research and behavioral strategies can inform the development, implementation, monitoring, and evaluation of theory-informed strategies to promote exclusive LPG use in future stove programs and research studies.

For peer review only - http://bmjopen.bmj.com/site/about/guidelines.xhtml

Keywords: cleaner fuels, behavior change communication, liquefied petroleum gas, adoption, household air pollution, behavioral theory

Acronyms:
BCC: Behavior Change Communication
BCI: Behavior Change Intervention
BCW: Behaviour Change Wheel
CD: Cooking Demonstration
COM-B: Capability, Opportunity, Motivation–Behaviour
FGD: Focus Group Discussion
HAP: Household Air Pollution
HAPIN: Household Air Pollution Intervention Network
IDI: In-Depth Interview
LPG: Liquefied Petroleum Gas
SUMS: Stove Use Monitoring Systems
TDF: Theoretical Domains Framework

Correspondence:
Kendra N. Williams, PhD
Division of Pulmonary and Critical Care Medicine
Johns Hopkins University
1830 E. Monument St Room 555
Baltimore, MD 21210
kendra.williams@jhu.edu

For peer review only

Strengths and Limitations of this Study:

- This article presents findings from theory-guided formative research and a protocol to monitor and reinforce exclusive use of liquefied petroleum gas (LPG) for cooking within the Household Air Pollution Network Intervention (HAPIN) trial in Guatemala, India, Peru, and Rwanda.
- Application of the Capability, Opportunity, Motivation–Behavior framework and Behavior Change Wheel facilitated the identification of context-specific influencers of fuel choice and enabled development of tailored behavioral strategies to promote exclusive LPG use.
- Monitoring stove use through observations, questionnaires, and temperature data loggers will allow us to examine and reinforce the near-exclusive LPG use necessary to realize potential air quality and health improvements in the main trial.
- The extensive behavioral monitoring and reinforcement package we outlined may not be feasible for replication in all contexts, and will not enable us to identify which components are necessary and sufficient to achieve exclusive LPG use.
- Behavior change and monitoring strategies presented here can be adapted for use in other programs and research studies aiming to promote exclusive use of cleaner fuels.

For peer review only

INTRODUCTION

Nearly three billion people worldwide use solid fuel (wood, charcoal, dung, crop residue, or coal) and kerosene for cooking, heating, and lighting.[1] Use of these fuels leads to high levels of household air pollution (HAP), resulting in negative impacts on health, environment, well-being, and climate.[2] Substitution of cleaner-burning fuels such as liquefied petroleum gas (LPG) has the potential to mitigate these negative outcomes.[3]

Stove programs and research studies have focused on improved cookstoves (e.g. rocket or vented chimney stoves[4-8]) and cleaner fuels (e.g. pellet[9], ethanol[10,11], and LPG[12,13]) to reduce exposure to fine particulate matter ($PM_{2.5}$) and carbon monoxide (CO) and subsequently improve health.[14] However, most report limited exposure reductions (post-intervention 24-48-hour mean $PM_{2.5}$ kitchen concentrations range from 120-280 μg/m^3 for cleaner fuel stoves and 290-410 μg/m^3 for improved solid fuel stoves) and uncertain health benefits.[15] One of the main reasons for this is the continued use of solid fuel stoves alongside cleaner fuel stoves—a practice known as stove stacking.[14] Models indicate that just one hour of traditional stove use per week can raise exposure to $PM_{2.5}$ and CO above the World Health Organization (WHO) recommended interim target of 35 μg/m^3 for annual mean $PM_{2.5}$ concentration.[16,17] To reach this target, many programs are shifting away from improved solid fuel stoves towards promoting exclusive use of cleaner fuels. Equally important is the dis-adoption of solid fuel stoves.[14,18,19]

Cleaner fuel options for low- and middle-income countries (LMICs) include LPG, electricity, piped natural gas, alcohol, and biogas. However, electric stoves are not yet a viable option in regions with small, unreliable electric grids, piped natural gas is not widely available, alcohol

For peer review only - http://bmjopen.bmj.com/site/about/guidelines.xhtml

fuel supply is typically limited, and biogas is a high-maintenance option for rural settings.[20,21] LPG is a viable, scalable cleaner fuel option, however there are significant barriers to sustained, exclusive LPG use in LMICs.[20] The primary barrier is cost: poor families often cannot afford to purchase LPG cooking equipment or refill gas cylinders.[20,22-24] Another major barrier is access, especially in rural areas where the LPG supply infrastructure is limited.[20,24] At the household level, other factors play a role, including perceptions that traditional foods prepared with a solid fuel stove taste better[25-27], and that two-burner LPG stoves cannot accommodate cooking large quantities of food.[28] Finally, fear that LPG stoves are dangerous may impede adoption.[20]

While overcoming behavioral barriers is critical to achieving long-term use of cleaner cookstoves and fuel, few programs and research studies have integrated behavioral components into their campaigns, instead focusing on short-term adoption of the new technology.[14,18] Those that did include behavioral training often lacked theoretically-grounded and context-specific formative research on behavioral factors influencing exclusive use.[18] Analytical frameworks and conceptual models such as the Risks, Attitudes, Norms, Abilities, and Self-regulation (RANAS) model[29], Capability, Opportunity, Motivation-Behaviour (COM-B) Model, and Behaviour Change Wheel (BCW) can guide the development and implementation of behavioral interventions[30-32], but have not been widely used to promote sustained, exclusive use of cleaner cookstoves and fuels.[33-36]

We sought to overcome these behavioral barriers within the Household Air Pollution Intervention Network (HAPIN) trial. The HAPIN trial aims to measure the effect of an LPG cooking intervention on HAP and health among study populations in Guatemala, India, Peru, and

For peer review only - http://bmjopen.bmj.com/site/about/guidelines.xhtml

Rwanda. Using a randomized controlled design, HAPIN will enroll 800 pregnant women (nine to <20 weeks gestational age) and up to 200 older adult women residing in the same homes in each country. Participants in the intervention group will receive an LPG stove and two LPG cylinders, approximately 18 months of free LPG deliveries, stove repairs as needed, and continuous cooking behavior-change support. Primary outcomes are low birth weight, stunting and severe pneumonia in children less than one year of age, and blood pressure in older women.[37]

Achieving exclusive LPG use and dis-adoption of solid fuel stoves are essential to reduce HAP exposures within the HAPIN trial. In this paper, we describe formative research guided by the COM-B, BCW, and Theoretical Domains Framework (TDF) to develop locally-adapted behavioral strategies for promoting exclusive LPG use.[31,32,38] We first present a comparison of key findings from formative research activities related to perceptions and use of LPG across the four research sites. We then discuss how we applied findings to develop behavioral interventions designed to achieve exclusive LPG use. We conclude with a protocol outlining the strategies we will use to monitor and reinforce LPG use in the main HAPIN trial.

METHODS

Guiding principles for behavioral strategies

The HAPIN research team formed a Behavioral and Economics Core (BEC) to address behavioral components of the trial. The BEC includes representatives from each participating country and health behavior experts who provide guidance. The BEC concluded that behavioral strategies would require adaptation to contextual differences of each site, but strategies should share a common set of guiding principles, including:

For peer review only - http://bmjopen.bmj.com/site/about/guidelines.xhtml

1. Provide appropriate training on proper use and maintenance of LPG stoves and equipment to ensure safe operation.
2. Address context-specific barriers and facilitators to sustained, exclusive use of LPG and dis-adoption of traditional solid fuel stoves.
3. Maximize exclusive LPG use and minimize use of solid fuels among intervention households.
4. Monitor solid fuel stove use and reinforce exclusive LPG use in intervention households that continue to use solid fuels for cooking
5. Avoid emphasizing potential health benefits of LPG to minimize the risk of introducing bias when participants report health outcomes.

Formative Research

Theoretical grounding

We used the COM-B and BCW to guide the design of formative research activities and to apply findings to the development of behavioral interventions. The COM-B Model is a behavioral system that provides a foundation for evaluating the capabilities, opportunities, and motivations that drive behavior, highlighting that a "behavioral diagnosis" must be understood to develop effective interventions.[32] The components of the COM-B map onto the theoretically-derived determinants of behavior from the TDF.[30] The TDF is comprised of 14 theoretical domain functions (e.g. knowledge, skills, attention and decision processes, beliefs about capabilities, intentions, goals, social influences, environmental context and resources) and 84 constructs (e.g. procedural knowledge, attention control, action planning, self-efficacy, material resources, social norms) synthesized from multiple theoretical models. The BCW includes nine intervention

For peer review only

functions (education, persuasion, incentivization, coercion, training, enablement, modeling, environmental restructuring, and restrictions) that can be applied to address gaps in identified capabilities, opportunities, and motivations to promote behavior change.[31,32] Using the COM-B, TDF, and BCW frameworks, we selected relevant domains and functions to develop behavioral strategies that are contextually specific (across HAPIN research sites) and grounded in theory.[32]

Study Sites

Formative research was conducted in Jalapa Department, Guatemala; Puno Province, Peru; Kayonza District, Eastern Province, Rwanda; and Nagapattinam and Kallakurichi (previously Villupuram) Districts in Tamil Nadu, India. Formative research surveys found LPG stove ownership in these regions to be 0% in Rwanda, 68% in Peru[39], 31% in Guatemala, and 57% in India. However, exclusive or primary use of LPG stoves was lower: 0% in Rwanda, 3.5% in Peru[39], 13% in Guatemala, and 29.5% in India.

In depth interviews, rapid assessments, and focus group discussions

In-depth interviews (IDI) were conducted using semi-structured interview guides, tailored for each research site. The following themes were covered:

1. Stoves owned/frequency of use
2. Preferred stoves for traditional dishes/beverages
3. Family influences on stove/fuel use
4. Temporal, seasonal, and circumstantial influences on stove choice
5. Perceived benefits/disadvantages of traditional stoves
6. Knowledge/perceptions of LPG stoves

For peer review only - http://bmjopen.bmj.com/site/about/guidelines.xhtml

7. Reasons for stove stacking
8. Fuel purchase/solid fuel collection practices
9. Perceived impact of LPG on daily life/household status
10. Cooking tasks/consumption patterns, including during pregnancy/after birth

In Rwanda, cooking demonstrations and food tasting tests were conducted prior to IDIs in participating homes who lacked exposure to LPG. Several focus group discussions (FGDs) were conducted in Rwanda to develop materials given minimal familiarity with LPG in the study area.

Behavior change materials were developed based on IDI and FGD findings by local teams. FGDs were then conducted with participants at all sites to review draft materials. Participants were asked to describe their understanding of the messages being conveyed, any barriers and facilitators to LPG use not captured, whether messages could be understood based solely on the pictures (given low literacy rates), and whether participants felt represented by the images. In India, FGDs aimed to identify a minimum set of information necessary for promoting exclusive LPG use among intervention households, which would minimize resentment and contamination bias in control households. Materials were modified based on FGD feedback.

Pilot studies with LPG cooking equipment

Following development of the behavior change materials, we conducted pilot studies to test these messages, among other study procedures.[40] Women in India (n=40), Rwanda (n=40), and Guatemala (n=60) were provided LPG stoves and free fuel for two months, along with behavior change messages. We tested the effectiveness of the behavioral messages by assessing $PM_{2.5}$

exposure reductions compared to baseline, the rate of exclusive LPG use monitored by temperature data loggers (Dots™) on stoves[41], and feedback from participants and field staff. The team in Rwanda conducted additional FGDs with pilot participants to revise behavior change materials. In Peru, behavioral messages were developed and tested with non-pregnant adult women in the Cardiopulmonary outcomes and Household Air Pollution (CHAP) trial[13]; messages specific to pregnant women and new mothers were assessed through additional interviews and FGDs.

Behavioral strategy development

After finalizing the behavioral messages, a questionnaire and instruction sheet were developed using the COM-B model and BCW. This will guide the implementation of messages as part of a larger behavior change strategy and will be used to monitor the effectiveness in achieving exclusive LPG use.

Ethics Approval

The formative research protocol was reviewed and approved by the Institutional Review Boards of Emory University (00089799); the Bloomberg School of Public Health, Johns Hopkins University (00007464); Asociación Beneficia PRISMA in Peru (CE3571.16); Sri Ramachandra Institute of Higher Education and Research (IEC-N1/16/JUL/54/49); the Indian Council of Medical Research–Health Ministry Screening Committee (5/8/4-30/(Env)/Indo-US/2016-NCD-I); Universidad del Valle de Guatemala (146-08-2016); the Guatemalan Ministry of Health National Ethics Committee (11-2016); the London School of Hygiene and Tropical Medicine

For peer review only - http://bmjopen.bmj.com/site/about/guidelines.xhtml

(11664-2); and the Rwandan National Ethics Committee (No.148/RNEC/2017). The HAPIN trial is registered with clinicaltrials.gov (NCT02944682).

Patient and public involvement

The formative research reported in this manuscript was explicitly designed to engage community members at all four research sites in the design of an LPG intervention and behavioral reinforcement package to be implemented in the main HAPIN trial. Community members were involved in the initial identification of messages for promoting exclusive LPG use, as well as the refinement of the materials and methods for delivering those messages.

RESULTS

Table 1 summarizes the formative research activities conducted in each research site.

Table 1. Formative research methods to design a behavioral intervention for the HAPIN trial

	Guatemala	India	Peru	Rwanda
Participant observations<br><br>Cooking demonstrations	Participant observations of cooking activities in 36 homes with LPG and wood stoves, 2-3 hours in each home	N/A*	N/A*	18 two-hour LPG cooking demonstrations and blind food tasting with non-LPG users (participants did not keep LPG stoves or cylinders)
In-Depth Interviews	18 interviews with women (primary cooks; 26-68 years of age) and 6 group interviews with 3 or 4 male participants	25 interviews, 11 in Nagapattinam and 14 in Kallakurichi (previously Villupuram) (23 female cooks, 2 men; 6 solid fuel users, 4 LPG users, 15 mixed fuel users; 21-65 years of age)	7 interviews (6 pregnant women, 1 new mother)	54 interviews with female primary cooks (14 LPG users, 22 non-LPG users, and repeat interviews with 18 of the same non-LPG users after an LPG cooking demonstration)

For peer review only - http://bmjopen.bmj.com/site/about/guidelines.xhtml

Key Informant Discussions	1 informal interview with LPG distributor in Jalapa and 1 informal interview with the stove manufacturer	Informal discussions with LPG distributors and managers, and local community members	Informal discussions with field staff native to Puno	12 informal interviews with local field staff who installed the LPG stove and delivered behavioral training during the pilot study
Focus Group Discussions (FGDs)	9 FGDs of 5-6 participants (51 women; 2 men)	Two informal social group discussions (one in each site) with local villagers	1 FGD, 7 participants (4 pregnant women, 3 new mothers)	5 FGDs to develop behavior change materials (4 participants per group; 18-68 years of age), 4 FGDs to refine materials with pilot participants (2 FGDs after 1-month of LPG use, 2 FGDs after 2-months of LPG use; women 18-33 years of age; 0-2 children per household; 3-7 participants per group)
LPG stove pilot study	Behavioral messages reviewed upon LPG stove installation and reinforced at LPG cylinder delivery visits in 60 households over a 3 month period	Behavioral messages delivered at LPG stove installation to the 20 pilot intervention households.	N/A (messages and materials piloted through CHAP study)[13]	Behavioral messages and materials delivered to 40 pilot study households.

* Participant observations and cooking demonstrations were not conducted in Peru or India given widespread awareness of LPG and previous research in these areas.[28]

For peer review only

Formative research results

We identified nine main themes that influence exclusive LPG use: 1) Perceived disadvantages of solid fuel stoves, 2) Family influences on cooking decisions, 3) Traditional cookware and stoves on which they are used, 4) Traditional foods and preferences for stoves used to prepare them, 5) Other non-food related reasons for cooking, 6) Heating needs, 7) Previous awareness and

For peer review only - http://bmjopen.bmj.com/site/about/guidelines.xhtml

experience with LPG, 8) Safety concerns, and 9) Cost and availability of LPG. We provide a brief description of the themes below; specific sub-themes are summarized in Table 2.

Table 2. Summary of qualitative findings according to identified themes across study sites.

	Guatemala	India	Peru	Rwanda
1. Perceived disadvantages of solid fuel stoves				
Smoke is physically irritating	X	X	X	X
Solid fuel stoves dirty kitchens, cookware, clothes, and hands	X	X	X	X
Collecting and cooking with solid fuels requires time and energy costs	X	X	X	X
Monetary costs of solid fuel	X			X
Fear of snakes and environmental hazards when collecting fuel	X	X	X	X
Difficulty collecting and lighting wet solid fuel		X	X	
2. Family influences on cooking practices				
Family complaints that food gets cold quickly with LPG	X		X	
Family complaints that food cooked with LPG lacks flavor			X	
Family preference for food cooked with LPG because food does not taste like smoke				X
Family preference for LPG because food cooks faster	X	X	X	X
Family perception that LPG represents modernity		X		X
Husbands believe smoke harms their wives, but not husbands who do not cook	X			
3. Cookware				
Belief that commonly used clay pots cannot be used on LPG stoves	X		X	
Large, flat griddle required for tortillas	X			
Large pots required to cook staple foods	X			X
Meat, fish, and vegetables commonly roasted on open fires				X
4. Traditional food				
Perception that some traditional dishes taste better when cooked with solid fuel	X		X	X
Preference to cook food with solid fuel for family festivities and special occasions		X	X	X
Preference to cook beans with solid fuel	X			X
5. Other stove uses				
Heating water for bathing and washing	X	X	X	X

For peer review only - http://bmjopen.bmj.com/site/about/guidelines.xhtml

Cooking food for animals			X	
Making alcoholic beverages				X
6. Home heating needs				
Warmth from traditional stove viewed as beneficial during cold months	X		X	
7. LPG awareness				
Active governmental LPG campaigns have achieved high LPG awareness		X	X	
Low LPG awareness in countries that lack governmental LPG campaigns	X			X
8. LPG fears and safety				
Fear of LPG leaks and explosions or fires	X		X	X
Fear of properly attaching regulator and hose to the LPG cylinder			X	X
Fear of LPG-related burns			X	X
Concerns for child safety	X		X	X
Mistrust of LPG providers	X		X	
9. LPG cost, supply, and distribution				
LPG refills perceived as expensive	X	X	X	X
Large and highly-regulated governmental LPG market		X		
Fewer governmental controls on LPG market	X		X	X
Lack of LPG sale points and delivery capability in study areas	X		X	X
Households are difficult to access (large distances between homes, lack of roads for transport)	X		X	

Reasons for dis-adopting solid fuel stoves

Participants identified several disadvantages of solid fuel stoves, which suggest potential reasons for dis-adoption of traditional stoves.

Family preferences for cooking practices

Many participants mentioned that family preferences influenced decisions about which stove to use for cooking tasks.

For peer review only - http://bmjopen.bmj.com/site/about/guidelines.xhtml

Cookware

In Guatemala, Peru, and Rwanda, participants raised concerns that LPG stoves would not accommodate the pots and cookware they needed to cook local staple foods.

Traditional food

All sites, except India, identified traditional foods that people preferred to prepare with solid fuel stoves. Participants in Peru reported preferring to make a steamed quinoa bread (*quispiño*) with the traditional stove. In Guatemala and Rwanda, participants preferred to cook beans on the open fire because they believed beans cooked more slowly on LPG stoves. Additionally, in Rwanda, *ugali*, or cassava bread, is difficult to make on an LPG stove because of the force required to stir the dough, which could cause the burner grate to break.

Other uses of the stove

Traditional stoves are often used for purposes other than family meals, such as heating water for bathing during cold months. In Peru, people also commonly cook food for pigs and dogs. In Rwanda, open fires are sometimes used to make sorghum beer in large pots.

Home heating needs

Warmth emanating from the traditional stove was valued during cold months in Guatemala and Peru, and to a lesser extent in India. In Guatemala, participants used the traditional stove for space heating but said they would forgo this if they had free LPG. In Peru, participants described using extra layers of clothing instead of lighting their traditional stove for heat.

For peer review only

LPG awareness

In Peru and India, where governmental campaigns are actively promoting LPG nationwide, LPG awareness was much higher than in Guatemala and Rwanda, where no national LPG campaigns currently exist. Owning an LPG stove in India was considered highly aspirational.

LPG fears and safety

Participants reported some fears and concerns about LPG stove and cylinder safety, such as leaks, explosions, burns, and child safety. Several participants in Peru and Guatemala reported a lack of trust in the safety and reliability of products from some LPG companies. In India, participants' concerns about the safety of LPG were described as minimal and acceptable in light of other LPG benefits.

LPG cost, supply, and distribution

LPG refill costs were major barriers in all sites. Distance and inaccessibility of households also limited LPG cylinder refills. While the LPG market in India is extensive and highly regulated, there are fewer governmental controls and LPG sale points in Guatemala, Rwanda, and Peru.

Developing behavioral messages for the HAPIN trial

We mapped formative research findings onto the COM-B and TDF domains and developed behavioral messages to address identified themes and domains (Table 3). Factors related to capabilities and skills will be addressed using how-to materials and training, whereas factors related to motivation will be targeted with appeals to emotions such as trust, security, and conscious decision-making. Factors related to opportunity and context address physical

For peer review only

opportunities (providing prompt gas delivery and stove repair) as well as social opportunities (educating other members in the home to use the LPG stove) will be integrated into trial procedures.

Using the BCW, we identified seven intervention functions we will use to deliver messages that might lead to exclusive LPG use: *education* to increase knowledge and confidence in safe LPG use, *persuasion* to promote positive feelings about LPG benefits, *training* to enable LPG use to meet household needs, *environmental restructuring* to situate the LPG stove in kitchens that are free of smoke, *modeling* LPG stove use through hands-on training such as demonstrations of stove operation, *incentivization* by providing free LPG gas, and *enablement* by providing prompt LPG delivery and stove repairs. Because behavioral reinforcement visits are intended to be positive reinforcements and are not meant to be coercive or to induce negative emotions, two intervention functions, *restriction* and *coercion*, are not pertinent.

For peer review only

For peer review only - http://bmjopen.bmj.com/site/about/guidelines.xhtml

Table 3. Themes, behavioral messages, and strategies based on the Behavior Change Wheel developed during formative research for the HAPIN trial

Themes	Behavioral messages	COM-B/TDF domain[1]	Strategies and *related Intervention Functions*
1. Perceived disadvantages of solid fuel	Using gas prevents discomfort (by reducing smoke)<br>Gas can be used in all seasons/weather<br>Using gas is easy<br>Gas eliminates smoke in the home<br>Gas keeps hands, clothes, pots and kitchens cleaner<br>With gas you do not need to collect or buy solid fuel<br>Gas will not make holes in thatch/aluminum roof	*Motivation*/reinforcement; emotions; optimism; beliefs about consequences<br><br>*Opportunity*/environmental context and resources	• Emphasize disadvantages of traditional stoves to encourage dis-adoption<br>• *Education; Persuasion; Environmental restructuring*
2. Family influences	Tips for addressing concerns of household members<br>Tips for addressing concerns of friends/neighbors<br>You can keep foods hot, or reheat quickly, after cooking them with gas<br>Using gas saves money and time	*Motivation*/emotion; beliefs about capabilities; optimism<br><br>*Opportunity*/social influences	• Target behavioral interventions to all household members, not just primary cooks<br>• *Education; Persuasion; Enablement*
3. Cookware	Using clay and other pots on the gas stove<br>How to cook large quantities of food with gas<br>How to roast on an LPG stove	*Capability*/knowledge; skills	• Stove use demonstrations<br>• Guatemala and Rwanda: Provide cookware to enable typical cooking behaviors<br>• *Training; Modelling*
4. Traditional food	It is possible to cook beans on an LPG stove<br>How to cook traditional dishes with gas<br>How to enhance food flavor without solid fuel<br>How to make beer on an LPG stove<br>Practice makes perfect	*Capability*/knowledge; skills<br><br>*Motivation*/reinforcement; intentions; beliefs about capabilities; optimism	• Guatemala and Rwanda: Encourage soaking beans<br>• Rwanda: Emphasize removing large pots from stove for forceful stirring<br>• *Education; Persuasion; Training*
5. Other stove uses	Everything can be done with gas	*Motivation*/goals; reinforcement; intentions; beliefs about capabilities	• Reassure households that LPG will be provided to meet all household cooking needs

For peer review only

		Opportunity/environmental context and resources	• *Education; Persuasion; Incentivization; Environmental restructuring*
6. Home heating needs	How to stay warm when cooking with gas	*Motivation*/reinforcement; intentions; beliefs about consequences	• Emphasize that no stove should be used for heating home<br>• Emphasize other LPG benefits as trade-offs for lack of heat<br>• *Education; Persuasion*
7. LPG awareness	How to use LPG stove (turn off, turn on, open and close the gas)<br>How to regulate the flame, to prevent burning food and to save gas<br>How to clean stove	*Capability*/knowledge; skills; memory, attention and decision processes	• Hands-on training on stove operation<br>• *Education; Training*
8. LPG fears and safety	Gas is natural, like wood; the smell added to it is unpleasant to alert leaks, but not toxic<br>How to avoid burns<br>Child safety<br>If used correctly, LPG stoves are completely safe<br>How to check for and respond to a leak (soapy water)<br>How to change the cylinder<br>Explaining reasons why LPG brand can be trusted<br>Millions of people use LPG stoves with no problems<br>Who to call if there is a problem<br>Where/how to get technical support<br>How to store stove and gas cylinders properly	*Capability*/knowledge; skills; reinforcement; memory, attention and decision processes<br><br>*Motivation*/emotion; beliefs about capabilities	• Provide training on gas safety; provide phone numbers for project staff if leak detected or stove in need of repair; respond to household fears around gas use<br>• *Education; Persuasion; Training; Environmental restructuring; Enablement*
9. LPG costs, supply, and distribution	Anticipating when gas will run out (cylinder check)<br>What to do when you need a gas refill (including when and who to call)<br>Security measures to prevent theft	*Capability*/knowledge; skills; reinforcement; memory, attention and decision processes<br><br>*Opportunity*/environmental context and resources	• Provide phone numbers for project staff if need gas refill; at installation instruct on secure storage of stove and cylinders<br>• *Enablement; Education; Persuasion; Training; Environmental restructuring*

[1]Examples of theoretical domains are provided, but are not exhaustive

For peer review only - http://bmjopen.bmj.com/site/about/guidelines.xhtml

Protocol for delivering behavioral strategies during the HAPIN trial

Stove package and equipment

Free, unlimited supply of LPG will be provided to intervention arm participants to incentivize exclusive LPG use. To ensure constant supply (intervention function: environmental restructuring), two LPG cylinders will be provided. In Guatemala and Rwanda, the cylinders will have T-valve regulators with a flow switch that can be toggled to a second full tank when the first is empty. In India and Peru, families will be instructed to manually move the regulator between the cylinders. In Guatemala, the two cylinders will be installed outside the kitchen with a protective barrier. In Rwanda and Peru, cylinders will be installed inside, due to potential theft and freezing temperatures, respectively. Guatemala, Peru, and Rwanda will provide a three-burner stove and India will provide a two-burner stove, deemed to fulfill cooking needs during formative research. In Rwanda and India, tables will be provided for the LPG stove; in Peru and Guatemala, table-height stoves will be provided. To assure that traditional foods will be cooked on the LPG stove, the Guatemalan stove will include a griddle (*comal*) for cooking tortillas and households will receive a set of enamel pots. In Peru, households will be instructed to grease clay pots before using to prevent cracking. Households in Rwanda will be given a roasting appliance for grilling meats and vegetables.

Stove use pledge

When the LPG equipment is installed in intervention households, field staff will ask all household members to be present and will administer a verbal pledge. By completing the pledge, participants will affirm that they; a) understand the study goals of reducing smoke exposures and achieving exclusive LPG use, b) that any type of food can be cooked with LPG, c) that the LPG

For peer review only - http://bmjopen.bmj.com/site/about/guidelines.xhtml

stove should be used only for household cooking needs, d) that the stove/cylinder should not be sold or rented, e) that HAPIN staff are available to help with any challenges related to the LPG stove, and f) that all household members intend to use the LPG stove exclusively (intervention function: persuasion).

Stove installation and training

At the LPG stove installation visit, trained field technicians will provide training on: 1) lighting/adjusting the gas flame, 2) cleaning/maintaining the stove, 3) detecting/responding to gas leaks, 4) requesting cylinder refills and stove/cylinder repairs, 5) safe handling/use of cylinders and regulators, 6) benefits of LPG, and 7) disadvantages of solid fuel. In India, authorized technicians will collaborate with HAPIN staff to provide this training. In Rwanda, households will be required to pass a certification exam, demonstrating their ability to correctly perform the steps for safe stove and cylinder use before LPG stove installation (intervention functions: education and training)

Printed materials (calendars, booklets, pamphlets, posters)

At stove installation, study staff in Guatemala, Peru, and Rwanda will use a flipchart to deliver behavioral messages to participants. Participants in Guatemala, Rwanda, and Peru will also receive a printed guide, calendar, and/or poster containing pictorial and written representations of the behavioral messages to keep in their homes. In India, a flyer showing that a range of potential cooking tasks should be performed with LPG instead of the traditional stove will be left with households. Because LPG is highly aspirational and increasingly available through governmental

For peer review only - http://bmjopen.bmj.com/site/about/guidelines.xhtml

programs in India, printed materials on LPG benefits and traditional stove disadvantages will not be given to households to minimize unintended dissemination to control households.

Videos

In Guatemala and Rwanda, videos on safe stove and cylinder use, how to check for and respond to a gas leak, cleaning the gas stove, and cooking beans and other local dishes will be shown on a tablet to participants. Videos prepared in Rwanda will feature testimonials from both male and female LPG users, given formative research findings that men have a large influence over household decision-making.

Monitoring and reinforcing LPG use during the HAPIN trial

The following sections outline how we will monitor behavioral strategy effectiveness to achieve exclusive LPG use and how we will identify households that continue to use solid fuel for behavioral reinforcement visits.

Stove use monitoring

Temperature data loggers known as Dots™ (Geocene, Vallejo, CA, USA) will be installed on all solid fuel stoves in intervention households.[41] The Dots™ data loggers will be placed near or within the combustion zone to provide clear temperature signals at five-minute sampling intervals. Using a mobile application, field teams will download data from each Dot™ every two weeks to be analyzed on a secure cloud-based server. A deterministic algorithm will be used to identify rapid, sustained temperature increases, which will be flagged as traditional stove use events (Figure 1). Every week, local field staff, with periodic oversight by the BEC, will review

For peer review only - http://bmjopen.bmj.com/site/about/guidelines.xhtml

households with flagged traditional stove use events based on the Dot™ data. They will use this data to schedule reinforcement visits, as described below.

Observations of traditional stove use

Intervention households may build new makeshift fires that are not monitored by a temperature sensor or may remove the sensors from monitored stoves if they want to cook with solid fuel. Therefore, we will incorporate direct observations of traditional stove use into study activities. Field staff will conduct these observations at least once and up to three times per month in all intervention households. Using a checklist, staff will look for signs of recent traditional stove use, such as use during the visit, fresh ashes, hot embers, stoves that are warm to the touch, fresh blackening on walls, or lingering smoke.

Data tracking

Monthly LPG use will be monitored by LPG delivery staff, based on the frequency of refills provided to households. In households using less LPG than average, staff will assess whether supplemental solid fuel stove use is occurring. In households with high LPG usage, staff will confirm that the LPG stove is being used properly, i.e. not shared with neighbors, not used to prepare food for sale, appropriate flame settings to avoid fuel waste, and using lids. In Guatemala, Rwanda, and Peru, HAPIN staff will deliver LPG cylinder refills. In India, local LPG companies will deliver LPG with oversight from HAPIN staff.

LPG use reinforcement visits

Field staff will review the Dot™ data, observations, and LPG data described above to identify households using solid fuel stoves. Within one week of identifying the household, field staff will

For peer review only - http://bmjopen.bmj.com/site/about/guidelines.xhtml

visit to reinforce dis-adoption of the solid fuel stove and promote exclusive LPG use. A questionnaire will be administered to elicit concerns or challenges related to the LPG stove, allowing field staff to address their specific problems. For example, if participants mention that they cannot cook traditional dishes on the LPG stove, the field technician may show a how-to video or explain how to cook that dish using LPG. If participants are anxious about switching the valve between LPG cylinders, the staff will demonstrate the process and coach the participant to perform the procedure. In Guatemala, behavioral staff will observe cooking and conduct demonstrations with traditional stove users. In Rwanda, LPG testimonial videos featuring local families will be used to demonstrate benefits of LPG use.

Questionnaire on perceptions of LPG

A questionnaire on household perceptions of the LPG stove will be administered twice during pregnancy and twice after childbirth with intervention households. The questionnaire, based on the COM-B and BCW will uncover additional barriers and facilitators related to LPG use that could be incorporated into behavioral messaging as the main trial progresses.

DISCUSSION

We identified nine potential influencers of exclusive LPG use at the household level, including perceived disadvantages of solid fuel, family preferences, cookware, traditional foods, non-food related cooking, heating needs, LPG awareness, safety, and cost and availability of fuel. These factors are similar to those found by Puzzolo et al. (2016) in their systematic review of cleaner fuel use.[20] Our study is unique because we used formative research grounded in behavior change

For peer review only - http://bmjopen.bmj.com/site/about/guidelines.xhtml

theory to design behavioral strategies to promote exclusive use of LPG in intervention households and solid fuel stove dis-adoption for the HAPIN trial.

Too often, interventions assume that introduction of cleaner fuels and technologies alone will be enough to eliminate HAP exposure. However, without a clear understanding and targeted approach to address cooking behaviors, family dynamics, and environmental constraints, households often resume use of solid fuel stoves for some or most of their cooking needs.[14] Our research was guided by an overarching set of common principles generalizable across contexts, but also uncovered contextual differences requiring tailored behavioral approaches. All behavioral strategies are intended to increase LPG adoption among intervention households with some contextualization to local conditions (e.g. climate differences, cooking practices) during the HAPIN trial.

Achieving exclusive use of new cooking technologies requires that study participants abandon, or dis-adopt, the old cooking technology. Such dis-adoption has been used in behavioral intervention studies to change low-value practices or harmful behaviors.[38] Everett Rogers' diffusion of innovation theory suggests that households are more likely to abandon an old technology in favor of a new one when the new device has relative advantages over the old one, is compatible with local practices, and is not too complex to use.[42] During our formative phase, we provided 40-60 homes in Guatemala, India, and Rwanda with LPG stoves for two months to test acceptability, appropriateness, and feasibility of LPG stove and fuel use.[40] This initial phase enabled us to identify local perceptions of the relative advantages of LPG over traditional stoves, local practices that needed to be framed as compatible with LPG, and how to reduce complexity of the

For peer review only - http://bmjopen.bmj.com/site/about/guidelines.xhtml

LPG technology that we incorporated into training and behavior change strategies in each research site.[42]

Our formative research highlighted several areas that build upon efforts of previous cookstove trials. For example, self-reported stove use has been shown to over-estimate use of the improved or cleaner stove and under-estimate continued use of the solid fuel stove.[43] Other studies have used temperature data loggers to monitor the cleaner stove but did not monitor all solid fuel stoves in the home, which limits the ability to estimate stove stacking.[44] To better understand stove use and stacking behaviors, our study applies temperature data loggers on all traditional stoves with observations of traditional stove use at monthly home visits. Real time data summaries will allow continuous follow-up during the trial, flagging households using traditional stoves. Field staff will visit homes to troubleshoot potential LPG stove problems or other barriers and reinforce exclusive LPG use. Observations and responses to questionnaires on LPG perceptions and use will inform continuous adaptation to behavioral messages to maximize LPG adoption.

We designed our behavioral messaging to emphasize immediately visible disadvantages of cooking with solid fuels such as dirty kitchens and physical discomfort to encourage abandonment, based on our formative research that suggested these disadvantages were more tangible than long-term health effects. Other studies have also found focusing on health risks to be less effective.[28] Addressing context-specific fears and concerns, grounded in theory, may prove to be more effective than solely addressing capabilities, or skills training, on how to use the LPG stove. While skills training is essential for adoption of unfamiliar technologies,

For peer review only - http://bmjopen.bmj.com/site/about/guidelines.xhtml

additional behavior change messages that target motivations and opportunities among all household members may encourage a more complete household transition to exclusive LPG use. The TDF describes motivations, or social norms, as an essential part of designing behavioral interventions, and household members may either support or thwart the use of a new stove technology. Because the trial will provide free LPG, we will target opportunities by addressing environmental resources and context. This will assure that participants will be able to use the LPG stove for all purposes, including cooking animal fodder and brewing beer, which is uncommon when people pay for their own fuel.[20]

Cost remains one of the main drivers of cleaner fuel adoption.[20,22,23] Both monetary and time costs of obtaining cleaner fuel are frequent barriers to adoption.[23,39] In many rural areas, LPG cylinders are not delivered to homes, requiring families to travel long distances to procure fuel.[45] The HAPIN trial will provide 18 months of free fuel delivered to intervention households to overcome economic and transportation barriers and promote exclusive LPG use. Our formative research highlighted additional factors unrelated to cost that we hypothesize must also be addressed to achieve exclusive LPG use, such as reinforcing perceived disadvantages of cooking with solid fuel, addressing fears of LPG, fulfilling non-cooking needs for stove use such as heating and preparing animal fodder, and ensuring that LPG cooking is compatible with traditional foods.

Several potential limitations should be noted. First, we may have missed important contextual factors during our formative research. For example, in multi-family households, one LPG stove per household may not be sufficient to meet everyone’s needs. Additionally, positive behavioral

For peer review only - http://bmjopen.bmj.com/site/about/guidelines.xhtml

reinforcements may not be sufficient for intervention households that refuse to abandon solid fuel stoves. The complexity of changing cooking behaviors is one of the greatest challenges in stove adoption studies.[25,46,47] Second, our monitoring strategies may not accurately flag traditional stove use, which may result in unnecessary behavioral reinforcement visits to compliant households. Third, while we will track monthly LPG usage to assure that LPG households are requesting refills, LPG usage varies based on differences in household cooking tasks, family size, and other factors. Thus, we may incorrectly flag low LPG users for reinforcement. However, our extensive monitoring of stove use through observations, stove use questionnaires, and Dot™ data loggers will allow triangulation and offer insights into reasons for use and non-use of the LPG intervention over the 18-month trial. Lastly, our formative research, behavior change intervention, and monitoring plans are extensive and may not be feasible in all contexts. The HAPIN trial is not designed to determine which aspects of the intervention are critical for achieving exclusive LPG use, but rather to do everything possible to achieve exclusive use. Future research will be needed to test which components, i.e. cost removal, home delivery, stove use training, behavioral reinforcement, etc., are necessary and sufficient to achieve exclusive LPG use.

For peer review only

CONCLUSION

Achieving the highest possible exclusive LPG use among intervention households is essential for understanding the potential exposure reductions and health benefits that an LPG cooking intervention can provide. While our approach is more intensive than a real-world LPG promotion program, our formative research results provide valuable insights on how to develop, implement, monitor, and evaluate theory-informed behavioral strategies to promote LPG adoption and

For peer review only - http://bmjopen.bmj.com/site/about/guidelines.xhtml

exclusive use. Strategies for promoting and monitoring exclusive LPG use are important not only to understand the impact of LPG adoption within trials, but also to sustain use in broader programs and promotional campaigns. While the behavioral components of the intervention were designed in the context of the HAPIN trial, the methods and lessons learned may provide insights for achieving sustained, exclusive use of cleaner fuels when delivered programmatically at scale.

For peer review only

For peer review only - http://bmjopen.bmj.com/site/about/guidelines.xhtml

ACKNOWLEDGMENTS

The HAPIN trial is funded by the U.S. National Institutes of Health [cooperative agreement 1UM1HL134590] in collaboration with the Bill & Melinda Gates Foundation [OPP1131279].

A multidisciplinary, independent Data and Safety Monitoring Board (DSMB) appointed by the National Heart, Lung, and Blood Institute (NHLBI) monitors the quality of the data and protects the safety of patients enrolled in the HAPIN trial. NHLBI DSMB: Nancy R. Cook, Sc.D.; Stephen Hecht, Ph.D.; Catherine Karr, M.D., Ph.D.; Katie H. Kavounis, M.P.H.; Dong-Yun Kim, Ph.D.; Joseph Millum, Ph.D.; Lora A. Reineck, M.D., M.S.; Nalini Sathiakumar, M.D., Dr.P.H.; Paul K. Whelton, M.D.; Gail G. Weinmann, M.D.

Program Coordination: Gail Rodgers, M.D., Bill & Melinda Gates Foundation; Claudia L. Thompson, Ph.D. National Institute of Environmental Health Science (NIEHS); Mark J. Parascandola, Ph.D., M.P.H., National Cancer Institute (NCI); Danuta M. Krotoski, Ph.D. Eunice Kennedy Shriver National Institute of Child Health and Human Development (NICHD); Joshua P. Rosenthal, Ph.D. Fogarty International Center (FIC), Conception R. Nierras, Ph.D. NIH Office of Strategic Coordination Common Fund; Antonello Punturieri, M.D., Ph.D. and Barry S. Schmetter, National Heart, Lung, and Blood Institute (NHLBI).

The findings and conclusions in this report are those of the authors and do not necessarily represent the official position of the U.S. National Institutes of Health, Department of Health and Human Services, or the Bill and Melinda Gates Foundation.

For peer review only

Competing Interests:

The authors declare they have no actual or potential competing interests.

Contributors:

KNW and LMT led the writing of the manuscript. ZS and GR managed data collection and analysis in Rwanda. MH, ADA, and LMT managed data collection and analysis in Guatemala. GT and KB managed data collection and analysis in India. KNW, SAH, and WC managed data collection and analysis in Peru. AQ, EP, JP, TFC, JJM, and JPR provided overall guidance to study implementation. All authors contributed to the cross-site synthesis of findings, development of the study protocol, and writing and revision of the manuscript.

Data Sharing:

De-identified participant data are available upon reasonable request. Contact Kendra Williams (kendra.williams@jhu.edu) for Peru data, Lisa Thompson (lisa.thompson@emory.edu) for Guatemala data, Gurusamy Thangavel (thangavel@ehe.org.in) for India data, or Ghislaine Rosa (ghislaine.rosa@lshtm.ac.uk) for Rwanda data. For data re-use conditions, please contact Lance Waller (lwaller@emory.edu).

For peer review only

For peer review only - http://bmjopen.bmj.com/site/about/guidelines.xhtml

REFERENCES

1. World Health Organization. *Burning opportunity: Clean household energy for health, sustainable development, and wellbeing of women and children. (no. ISBN 978 92 4 156523 3, NLM classification: WA 754).* Geneva, Switzerland. www.who.int; 2016.

2. Martin WJ, Glass RI, Araj H, et al. Household air pollution in low- and middle-income countries: Health risks and research priorities. *PLOS Medicine*. 2013;10(6):e1001455.

3. Bruce N, Pope D, Rehfuess E, Balakrishnan K, Adair-Rohani H, Dora C. WHO indoor air quality guidelines on household fuel combustion: Strategy implications of new evidence on interventions and exposure-risk functions. *Atmos Environ*. 2015;106:451-457.

4. Romieu I, Riojas-Rodriguez H, Marron-Mares AT, Schilmann A, Perez-Padilla R, Masera O. Improved biomass stove intervention in rural Mexico: Impact on the respiratory health of women. *Am J Respir Crit Care Med*. 2009;180(7):649-656.

5. Schilmann A, Riojas-Rodriguez H, Ramirez-Sedeno K, Berrueta VM, Perez-Padilla R, Romieu I. Children's respiratory health after an efficient biomass stove (patsari) intervention. *Ecohealth*. 2015;12(1):68-76.

6. Smith KR, McCracken JP, Weber MW, et al. Effect of reduction in household air pollution on childhood pneumonia in Guatemala (RESPIRE): A randomised controlled trial. *Lancet*. 2011;378(9804):1717-1726.

For peer review only - http://bmjopen.bmj.com/site/about/guidelines.xhtml

7. Hanna R, Duflo E, Greenstone M. Up in smoke: The influence of household behavior on the long-run impact of improved cooking stoves. *American Economic Journal: Economic Policy*. 2016;8(1):80-114.

8. Bensch G, Peters J. The intensive margin of technology adoption - experimental evidence on improved cooking stoves in rural Senegal. *J Health Econ*. 2015;42:44.

9. Mortimer K, Ndamala CB, Naunje AW, et al. A cleaner burning biomass-fuelled cookstove intervention to prevent pneumonia in children under 5 years old in rural Malawi (the cooking and pneumonia study): A cluster randomised controlled trial. *Lancet*. 2017;389(10065):167-175.

10. Alexander D, Northcross A, Wilson N, et al. Randomized controlled ethanol cookstove intervention and blood pressure in pregnant Nigerian women. *Am J Respir Crit Care Med*. 2017;195(12):1629-1639.

11. Alexander DA, Northcross A, Karrison T, et al. Pregnancy outcomes and ethanol cook stove intervention: A randomized-controlled trial in Ibadan, Nigeria. *Environ Int*. 2018;111:152-163.

12. Jack DW, Asante KP, Wylie BJ, et al. Ghana randomized air pollution and health study (GRAPHS): Study protocol for a randomized controlled trial. *Trials*. 2015;16:420-015-0930-8.

13. Fandino-Del-Rio M, Goodman D, Kephart JL, et al. Effects of a liquefied petroleum gas stove intervention on pollutant exposure and adult cardiopulmonary outcomes (CHAP): Study protocol for a randomized controlled trial. *Trials*. 2017;18(1):518.

14. Quinn AK, Bruce N, Puzzolo E, et al. An analysis of efforts to scale up clean household energy for cooking around the world. *Energy Sustain Dev*. 2018;46:1.

For peer review only - http://bmjopen.bmj.com/site/about/guidelines.xhtml

15. Pope D, Bruce N, Dherani M, Jagoe K, Rehfuess E. Real-life effectiveness of "improved" stoves and clean fuels in reducing PM2.5 and CO: Systematic review and meta-analysis. *Environ Int*. 2017;101:7-18.

16. Johnson MA, Chiang RA. Quantitative guidance for stove usage and performance to achieve health and environmental targets. *Environ Health Perspect*. 2015;123(8):820-826.

17. World Health Organization (WHO). *WHO indoor air quality guidelines: Household fuel combustion.* www.who.int; 2014.

18. Goodwin NJ, O'Farrell SE, Jagoe K, et al. Use of behavior change techniques in clean cooking interventions: A review of the evidence and scorecard of effectiveness. *J Health Commun*. 2015;20(sup1):43-54.

19. Hanbury A, Farley K, Thompson C, Wilson PM, Chambers D, Holmes H. Immediate versus sustained effects: Interrupted time series analysis of a tailored intervention. *Implement Sci*. 2013;8:130-5908-8-130.

20. Puzzolo E, Pope D, Stanistreet D, Rehfuess EA, Bruce NG. Clean fuels for resource-poor settings: A systematic review of barriers and enablers to adoption and sustained use. *Environ Res*. 2016;146:218-234.

21. Smith KR. Changing paradigms in clean cooking. *EcoHealth*. 2015;12:196-199.

22. Abdulai MA, Afari-Asiedu S, Carrion D, et al. Experiences with the mass distribution of LPG stoves in rural communities of Ghana. *Ecohealth*. 2018;15(4):757-767.

For peer review only

For peer review only - http://bmjopen.bmj.com/site/about/guidelines.xhtml

23. Gould CF, Urpelainen J. LPG as a clean cooking fuel: Adoption, use, and impact in rural India. *Energ Policy*. 2018;122:395.

24. Ronzi S, Puzzolo E, Hyseni L, et al. Using photovoice methods as a community-based participatory research tool to advance uptake of clean cooking and improve health: The LPG adoption in Cameroon evaluation studies. *Soc Sci Med*. 2019;228:30-40.

25. Alam A, Tawale N, Patel A, Dibley MJ, Jadhao S, Raynes-Greenow C. Household air pollution intervention implications: Findings from qualitative studies and a field trial of clean cookstoves in two rural villages in India. *Int J Environ Res Public Health*. 2016;13(9):893. doi: 10.3390/ijerph13090893.

26. Rhodes EL, Dreibelbis R, Klasen EM, et al. Behavioral attitudes and preferences in cooking practices with traditional open-fire stoves in Peru, Nepal, and Kenya: Implications for improved cookstove interventions. *Int J Environ Res Public Health*. 2014;11(10):10310-10326.

27. Thompson LM, Hengstermann M, Weinstein JR, Diaz-Artiga A. Adoption of liquefied petroleum gas stoves in Guatemala: A mixed-methods study. *Ecohealth*. 2018;15(4):745-756.

28. Hollada J, Williams KN, Miele CH, Danz D, Harvey SA, Checkley W. Perceptions of improved biomass and liquefied petroleum gas stoves in Puno, Peru: Implications for promoting sustained and exclusive adoption of clean cooking technologies. *Int J Environ Res Public Health*. 2017;14(2):182.

For peer review only

For peer review only - http://bmjopen.bmj.com/site/about/guidelines.xhtml

29. Mosler HJ. A systematic approach to behavior change interventions for the water and sanitation sector in developing countries: A conceptual model, a review, and a guideline. *Int J Environ Health Res*. 2012;22(5):431-449.

30. Cane J, O'Connor D, Michie S. Validation of the theoretical domains framework for use in behaviour change and implementation research. *Implement Sci*. 2012;7:37-5908-7-37.

31. Michie S, Atkins L, West R. *The behaviour change wheel: A guide to designing interventions.* London: Silverback Publishing; 2014. www.behaviourchangewheel.com.

32. Michie S, van Stralen MM, West R. The behaviour change wheel: A new method for characterising and designing behaviour change interventions. *Implementation Science*. 2011;6(1):42.

33. Kwah KL, Fulton EA, Brown KE. Accessing national health service stop smoking services in the UK: A COM-B analysis of barriers and facilitators perceived by smokers, ex-smokers and stop smoking advisors. *Public Health*. 2019;171:123-130.

34. Gould GS, Bar-Zeev Y, Bovill M, et al. Designing an implementation intervention with the behaviour change wheel for health provider smoking cessation care for Australian indigenous pregnant women. *Implementation Science*. 2017;12(1):114.

35. Wodnik BK, Freeman MC, Ellis AS, Awino Ogutu E, Webb Girard A, Caruso BA. Development and application of novel caregiver hygiene behavior measures relating to food preparation, handwashing, and play environments in rural Kenya. *Int J Environ Res Public Health*. 2018;15(9):10.3390/ijerph15091994.

For peer review only - http://bmjopen.bmj.com/site/about/guidelines.xhtml

36. Carrion D, Dwommoh R, Tawiah T, et al. Enhancing LPG adoption in Ghana (ELAG): A factorial cluster-randomized controlled trial to enhance LPG adoption & sustained use. *BMC Public Health*. 2018;18(1):689-018-5622-3.

37. Household air pollution and health: A multi-country LPG intervention trial (HAPIN). ClinicalTrials.gov identifier: NCT02944682. U.S. National Library of Medicine Web site. https://clinicaltrials.gov/ct2/show/NCT02944682. Published October 26, 2016. Updated May 6, 2019.

38. Thompson LM, Diaz-Artiga A, Weinstein JR, Handley MA. Designing a behavioral intervention using the COM-B model and the theoretical domains framework to promote gas stove use in rural Guatemala: A formative research study. *BMC Public Health*. 2018;18(1):253.

39. Pollard SL, Williams KN, O'Brien CJ, et al. An evaluation of the Fondo de Inclusion Social Energetico program to promote access to liquefied petroleum gas in Peru. *Energy Sustain Dev*. 2018;46:82-93.

40. Liao J, McCracken JP, Piedrahita R, et al. The use of bluetooth low energy beacon systems to estimate indirect personal exposure to household air pollution. *J Expo Sci Environ Epidemiol*. 2019.

41. Geocene Inc. Geocene dots temperature loggers. *Vallejo, CA*. 2016;https://geocene.com;.

42. Rogers EM. *Diffusion of innovations.* 5th ed. New York: Free Press; 2003:551.

For peer review only - http://bmjopen.bmj.com/site/about/guidelines.xhtml

43. Lozier MJ, Sircar K, Christensen B, et al. Use of temperature sensors to determine exclusivity of improved stove use and associated household air pollution reductions in Kenya. *Environ Sci Technol*. 2016;50(8):4564-4571.

44. Ruiz-Mercado I, Lam N, Canuz E, Davila G, Smith KR. Low-cost temperature loggers as stove use monitors (SUMs). *Boiling Point*. 2008;55:16-18.

45. Asante KP, Afari-Asiedu S, Abdulai MA, et al. Ghana's rural liquefied petroleum gas program scale up: A case study. *Energy Sustain Dev*. 2018;46:94-102.

46. Mukhopadhyay R, Sambandam S, Pillarisetti A, et al. Cooking practices, air quality, and the acceptability of advanced cookstoves in Haryana, India: An exploratory study to inform large-scale interventions. *Glob Health Action*. 2012;5:1-13.

47. Hooper LG, Dieye Y, Ndiaye A, et al. Traditional cooking practices and preferences for stove features among women in rural Senegal: Informing improved cookstove design and interventions. *PLoS One*. 2018;13(11):e0206822.

For peer review only

FIGURE LEGEND

Figure 1. Geocene Dot^{TM} data from one household showing a flagged cooking event with a rapid temperature increase.

For peer review only

For peer review only - http://bmjopen.bmj.com/site/about/guidelines.xhtml

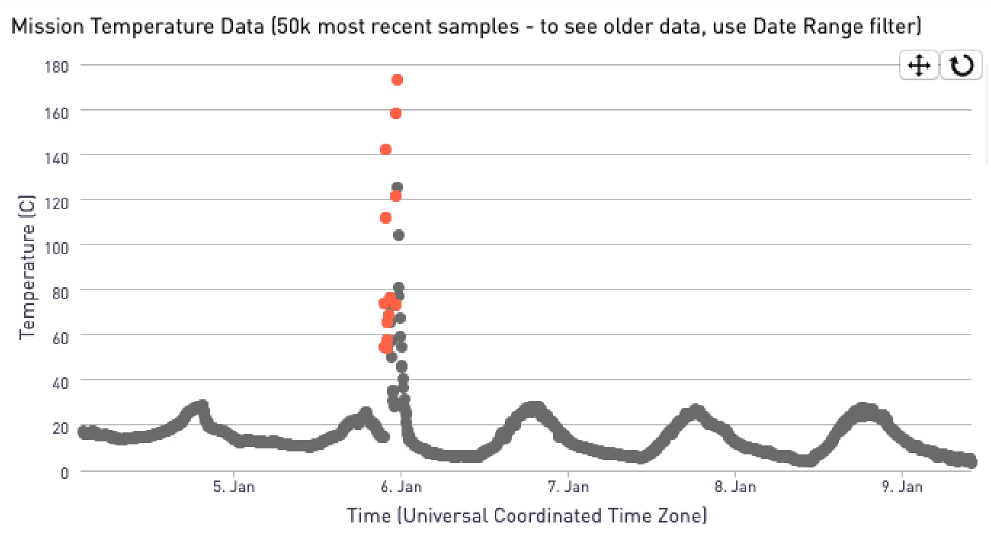

242x130mm (72 x 72 DPI)

BMJ Open

Designing a comprehensive behavior change intervention to promote and monitor exclusive use of liquefied petroleum gas stoves for the Household Air Pollution Intervention Network (HAPIN) trial

Journal:	*BMJ Open*
Manuscript ID	bmjopen-2020-037761.R1
Article Type:	Original research
Date Submitted by the Author:	02-Jun-2020
Complete List of Authors:	Williams, Kendra; Johns Hopkins School of Medicine, Division of Pulmonary and Critical Care Medicine; Johns Hopkins School of Medicine, Center for Global Non-Communicable Disease Research and Training<br>Thompson, Lisa; Emory University, Nell Hodgson Woodruff School of Nursing<br>Sakas, Zoe; London School of Hygiene and Tropical Medicine Faculty of Infectious and Tropical Diseases, Disease Control<br>Hengstermann, Mayari; Universidad del Valle de Guatemala Centro de Estudios en Salud<br>Quinn, Ashlinn; National Institutes of Health, Fogarty International Center<br>Díaz Artiga, Anaité; Universidad del Valle de Guatemala, Centro de Estudios en Salud<br>Thangavel, Gurusamy; Sri Ramachandra Medical College and Research Institute, Department of Environmental Health Engineering<br>Puzzolo, Elisa; University of Liverpool, Public Health and Policy<br>Rosa, Ghislaine; London School of Hygiene and Tropical Medicine Faculty of Infectious and Tropical Diseases, Department of Disease Control<br>Balakrishnan, Kalpana; Sri Ramachandra Medical College and Research Institute, Department of Environmental Health Engineering<br>Peel, Jennifer; Colorado School of Public Health, Environmental & Radiological Health Sciences<br>Checkley, William; Johns Hopkins School of Medicine, Division of Pulmonary and Critical Care Medicine; Johns Hopkins School of Medicine, Center for Global Non-Communicable Disease Research and Training<br>Clasen, Thomas; Emory University, Rollins School of Public Health<br>Miranda, J. Jaime; Universidad Peruana Cayetano Heredia, CRONICAS Center of Excellence in Chronic Diseases<br>Rosenthal, Joshua; National Institutes of Health, Fogarty International Center<br>Harvey, Steven; Johns Hopkins University Bloomberg School of Public Health, International Health, Social and Behavioral Interventions
**Primary Subject Heading**:	Global health
Secondary Subject Heading:	Qualitative research

Keywords:	PUBLIC HEALTH, QUALITATIVE RESEARCH, Community child health < PAEDIATRICS

SCHOLARONE™
Manuscripts

I, the Submitting Author has the right to grant and does grant on behalf of all authors of the Work (as defined in the below author licence), an exclusive licence and/or a non-exclusive licence for contributions from authors who are: i) UK Crown employees; ii) where BMJ has agreed a CC-BY licence shall apply, and/or iii) in accordance with the terms applicable for US Federal Government officers or employees acting as part of their official duties; on a worldwide, perpetual, irrevocable, royalty-free basis to BMJ Publishing Group Ltd ("BMJ") its licensees and where the relevant Journal is co-owned by BMJ to the co-owners of the Journal, to publish the Work in this journal and any other BMJ products and to exploit all rights, as set out in our licence.

The Submitting Author accepts and understands that any supply made under these terms is made by BMJ to the Submitting Author unless you are acting as an employee on behalf of your employer or a postgraduate student of an affiliated institution which is paying any applicable article publishing charge ("APC") for Open Access articles. Where the Submitting Author wishes to make the Work available on an Open Access basis (and intends to pay the relevant APC), the terms of reuse of such Open Access shall be governed by a Creative Commons licence – details of these licences and which Creative Commons licence will apply to this Work are set out in our licence referred to above.

Other than as permitted in any relevant BMJ Author's Self Archiving Policies, I confirm this Work has not been accepted for publication elsewhere, is not being considered for publication elsewhere and does not duplicate material already published. I confirm all authors consent to publication of this Work and authorise the granting of this licence.

For peer review only

Designing a comprehensive behavior change intervention to promote and monitor exclusive use of liquefied petroleum gas stoves for the Household Air Pollution Intervention Network (HAPIN) trial

Kendra N. WILLIAMS[1,2], Lisa M. THOMPSON[3] (co-first), Zoe SAKAS[4], Mayari HENGSTERMANN[5], Ashlinn QUINN[6], Anaité DIAZ ARTIGA[5], Gurusamy THANGAVEL[7], Elisa PUZZOLO[8], Ghislaine ROSA[4], Kalpana BALAKRISHNAN[7], Jennifer PEEL[9], William CHECKLEY[1,2], Thomas F. CLASEN[10], J. Jaime MIRANDA[11], Joshua P. ROSENTHAL[6], Steven A. HARVEY[12], and the HAPIN Investigators[†]

Order and affiliations of authors

1. Division of Pulmonary and Critical Care Medicine, School of Medicine, Johns Hopkins University
2. Center for Global Non-Communicable Disease Research and Training, School of Medicine, Johns Hopkins University, Baltimore, MD 21287
3. Nell Hodgson Woodruff School of Nursing, Emory University, Atlanta, GA 30322
4. Department of Disease Control, Faculty of Infectious and Tropical Diseases, London School of Hygiene & Tropical Medicine, London, UK
5. Centro de Estudios en Salud, Universidad del Valle de Guatemala, Guatemala City, Guatemala
6. Fogarty International Center, National Institutes of Health, Bethesda MD 20892*
7. Center for Advanced Research on Air Quality, Climate and Health, Department of Environmental Health Engineering, Sri Ramachandra Institute for Higher Education and Research, India
8. Department of Public Health and Policy, University of Liverpool, Liverpool, UK
9. Department of Environmental & Radiological Health Sciences, Colorado School of Public Health, Colorado State University, Fort Collins, CO 80523
10. Rollins School of Public Health, Emory University, Atlanta, GA 30322
11. CRONICAS Center of Excellence in Chronic Diseases, Universidad Peruana Cayetano Heredia, Lima, Peru
12. Department of International Health, Social and Behavioral Interventions Program, Johns Hopkins Bloomberg School of Public Health, Baltimore, MD 21205

[†] Household Air Pollution Intervention Network (HAPIN) trial Investigators:

Vigneswari Aravindalochanan, Kalpana Balakrishnan, Dana Boyd Barr, Vanessa Burrowes, Alejandra Bussalleu, Devan Campbell, Eduardo Canuz, Adly Castañaza, Howard Chang, William Checkley, Yunyun Chen, Marilú Chiang, Maggie L. Clark, Thomas Clasen, Rachel Craik, Mary Crocker, Victor Davila-Roman, Lisa de las Fuentes, Oscar De León, Anaité Diaz-Artiga, Ephrem Dusabimana, Lisa Elon, Juan Gabriel Espinoza, Irma Sayury Pineda Fuentes, Sarada Garg, Dina Goodman, Savannah Gupton, Meghan Hardison, Stella Hartinger, Steven A.

For peer review only - http://bmjopen.bmj.com/site/about/guidelines.xhtml

Harvey, Mayari Hengstermann, Phabiola M. Herrera, Shakir Hossen, Penelope Howards, Lindsay Jaacks, Shirin Jabbarzadeh, MichaelA.Johnson, Abigail Jones, Katherine Kearns, Miles Kirby, Jacob Kremer, Margaret A. Laws, Pattie Lenzen, Jiawen Liao, Amy E. Lovvorn, Fiona Majorin, Eric McCollum, John McCracken, Julia N. McPeek, Rachel Meyers, J. Jaime Miranda, Erick Mollinedo, Lawrence Moulton, Krishnendu Mukhopadhyay, Luke Naeher, Abidan Nambajimana, Durairaj Natesan, Florien Ndagijimana, Azhar Nizam, Jean de Dieu Ntivuguruzwa, Aris Papageorghiou, Jennifer Peel, Ricardo Piedrahita, Ajay Pillarisetti, Naveen Puttaswamy, Elisa Puzzolo, Ashlinn K. Quinn, Sarah Rajkumar, Usha Ramakrishnan, Rengaraj Ramasami, Davis Reardon, Ghislaine Rosa, Joshua P. Rosenthal, Barry Ryan, Sudhakar Saidam, Zoe Sakas, Sankar Sambandam, Jeremy A. Sarnat, Suzanne Simkovich, Sheela S. Sinharoy, Kirk R. Smith, Kyle Steenland, Damien Swearing, Gurusamy Thangavel, Lisa M. Thompson, Ashley Toenjes, Lindsay Underhill, Jean Damascene Uwizeyimana, Viviane Valdes, Kayla Valentine, Amit Verma, Lance Waller, Megan Warnock, Kendra N. Williams, Wenlu Ye, Bonnie Young

For peer review only

For peer review only - http://bmjopen.bmj.com/site/about/guidelines.xhtml

STRUCTURED ABSTRACT:

Introduction: Increasing use of cleaner fuels, such as liquefied petroleum gas (LPG), and abandonment of solid fuels is key to reducing household air pollution and realizing potential health improvements in low-income countries. However, achieving exclusive LPG use in households unaccustomed to LPG requires substantial behavior change. We conducted theory-grounded formative research to identify contextual factors influencing cooking fuel choice to guide the development of behavioral strategies for the Household Air Pollution Intervention Network (HAPIN) trial. The HAPIN trial will assess the impact of exclusive LPG use on air pollution exposure and health of pregnant women, older adult women, and infants under one year of age in Guatemala, India, Peru, and Rwanda.

Methods: Using the Capability, Opportunity, Motivation–Behavior (COM-B) framework and Behavior Change Wheel (BCW) to guide formative research, we conducted in-depth interviews, focus group discussions, observations, key informant interviews, and pilot studies to identify key influencers of cooking behaviors in the four countries. We used these findings to develop behavioral strategies likely to achieve exclusive LPG use in the HAPIN trial.

Results: We identified nine potential influencers of exclusive LPG use, including perceived disadvantages of solid fuels, family preferences, cookware, traditional foods, non-food related cooking, heating needs, LPG awareness, safety, and cost and availability of fuel. Mapping formative findings onto the theoretical frameworks, behavioral strategies for achieving exclusive LPG use in each research site included free fuel deliveries, locally-acceptable stoves and equipment, hands-on training, and printed materials and videos emphasizing relevant messages. In the HAPIN trial we will monitor and reinforce exclusive LPG use through temperature data loggers, LPG delivery tracking, in-home observations, and behavioral reinforcement visits.

Conclusion: Our formative research and behavioral strategies can inform the development, implementation, monitoring, and evaluation of theory-informed strategies to promote exclusive LPG use in future stove programs and research studies.

For peer review only

For peer review only - http://bmjopen.bmj.com/site/about/guidelines.xhtml

Keywords: cleaner fuels, behavior change communication, liquefied petroleum gas, adoption, household air pollution, behavioral theory

Acronyms:
BCC: Behavior Change Communication
BCI: Behavior Change Intervention
BCW: Behaviour Change Wheel
CD: Cooking Demonstration
COM-B: Capability, Opportunity, Motivation–Behaviour
FGD: Focus Group Discussion
HAP: Household Air Pollution
HAPIN: Household Air Pollution Intervention Network
IDI: In-Depth Interview
LPG: Liquefied Petroleum Gas
SUMS: Stove Use Monitoring Systems
TDF: Theoretical Domains Framework

Correspondence:
Kendra N. Williams, PhD
Division of Pulmonary and Critical Care Medicine
Johns Hopkins University
1830 E. Monument St Room 555
Baltimore, MD 21210
kendra.williams@jhu.edu

For peer review only

Strengths and Limitations of this Study:

- Application of the Capability, Opportunity, Motivation–Behavior framework and Behavior Change Wheel facilitated the identification of context-specific influencers of fuel choice.
- The theory-guided formative research methods enabled the development of tailored behavioral strategies to promote exclusive use of liquefied petroleum gas (LPG).
- Our formative research did not consider market forces or costs of LPG given the intent to inform a trial in which LPG will be delivered for free.
- The extensive behavioral monitoring and reinforcement protocol may not be feasible for replication in all contexts.

For peer review only

For peer review only - http://bmjopen.bmj.com/site/about/guidelines.xhtml

INTRODUCTION

Nearly three billion people worldwide use solid fuel (wood, charcoal, dung, crop residue, or coal) and kerosene for cooking, heating, and lighting.[1] Use of these fuels leads to high levels of household air pollution (HAP), resulting in negative impacts on health, environment, well-being, and climate.[2] Substitution of cleaner-burning fuels such as liquefied petroleum gas (LPG) has the potential to mitigate these negative outcomes.[3]

Stove programs and research studies have focused on improved cookstoves (e.g. rocket or vented chimney stoves[4-8]) and cleaner fuels (e.g. pellet[9], ethanol[10,11], and LPG[12,13]) to reduce exposure to fine particulate matter ($PM_{2.5}$) and carbon monoxide (CO) and subsequently improve health.[14] However, most report limited exposure reductions (post-intervention 24-48-hour mean $PM_{2.5}$ kitchen concentrations range from 120-280 $\mu g/m^3$ for cleaner fuel stoves and 290-410 $\mu g/m^3$ for improved solid fuel stoves) and uncertain health benefits.[15] One of the main reasons for this is the continued use of solid fuel stoves alongside cleaner fuel stoves—a practice known as stove stacking.[14] Models indicate that just one hour of traditional stove use per week can raise exposure to $PM_{2.5}$ and CO above the World Health Organization (WHO) recommended interim target of 35 $\mu g/m^3$ for annual mean $PM_{2.5}$ concentration.[16,17] To reach this target, many programs are shifting away from improved solid fuel stoves towards promoting exclusive use of cleaner fuels. Equally important is the abandonment of solid fuel stoves.[14,18,19]

Cleaner fuel options for low- and middle-income countries (LMICs) include LPG, electricity, piped natural gas, alcohol, and biogas. However, electric stoves are not yet a viable option in regions with small, unreliable electric grids, piped natural gas is not widely available, alcohol

For peer review only - http://bmjopen.bmj.com/site/about/guidelines.xhtml

fuel supply is typically limited, and biogas is a high-maintenance option for rural settings.[20,21] LPG is a viable, scalable cleaner fuel option, however there are significant barriers to sustained, exclusive LPG use in LMICs.[20] The primary barrier is cost: poor families often cannot afford to purchase LPG cooking equipment or refill gas cylinders.[20,22-24] Another major barrier is access, especially in rural areas where the LPG supply infrastructure is limited.[20,24] Markets that assure adequate supply to meet household demand are a critical need.[25-28] At the household level, other factors play a role, including perceptions that traditional foods prepared with a solid fuel stove taste better[29-31], and that two-burner LPG stoves cannot accommodate cooking large quantities of food.[32] Finally, fear that LPG stoves are dangerous may impede adoption.[20]

While overcoming behavioral barriers is critical to achieving long-term use of cleaner cookstoves and fuels, programs and research studies that have integrated behavioral components into their campaigns often lack theoretically-grounded and context-specific formative research on behavioral factors influencing exclusive use.[14,18,33] Analytical frameworks and conceptual models such as the Risks, Attitudes, Norms, Abilities, and Self-regulation (RANAS) model[34], Capability, Opportunity, Motivation-Behaviour (COM-B) Model, and Behaviour Change Wheel (BCW) can guide the development and implementation of behavioral interventions[35-37], but have not been widely used to promote sustained, exclusive use of cleaner cookstoves and fuels.[38-41]

We sought to overcome these barriers within the Household Air Pollution Intervention Network (HAPIN) trial.[42] The HAPIN trial aims to measure the effect of an LPG cooking intervention on HAP and health among study populations in Guatemala, India, Peru, and Rwanda. Using a randomized controlled design, HAPIN will enroll 800 pregnant women (nine to <20 weeks

For peer review only - http://bmjopen.bmj.com/site/about/guidelines.xhtml

gestational age) and up to 200 older adult women residing in the same homes in each country. Participants in the intervention group will receive an LPG stove and two LPG cylinders, approximately 18 months of free LPG deliveries, stove repairs as needed, and continuous cooking behavior-change support. Primary outcomes are low birth weight, stunting and severe pneumonia in children less than one year of age, and blood pressure in older women.[42,43]

Achieving exclusive LPG use and abandonment of solid fuel stoves are essential to reduce HAP exposures within the HAPIN trial. In this paper, we describe formative research guided by the COM-B, BCW, and Theoretical Domains Framework (TDF) to develop locally-adapted behavioral strategies for promoting exclusive LPG use within the HAPIN trial.[36,37,44] We first present a comparison of key findings from formative research activities related to perceptions and use of LPG across the four research sites. We then discuss how we applied findings to develop behavioral interventions designed to achieve exclusive LPG use. We conclude with a protocol outlining the strategies we will use to monitor and reinforce LPG use in the main HAPIN trial.

METHODS

Guiding principles for behavioral strategies

The HAPIN research team formed a Behavioral and Economics Core (BEC) to address behavioral components of the trial. The BEC includes representatives from each participating country and health behavior experts who provide guidance. The BEC concluded that behavioral strategies would require adaptation to contextual differences of each site, but strategies should share a common set of guiding principles, including:

For peer review only - http://bmjopen.bmj.com/site/about/guidelines.xhtml

1. Provide appropriate training on proper use and maintenance of LPG stoves and equipment to ensure safe operation.
2. Address context-specific barriers and facilitators to sustained, exclusive use of LPG and abandonment of traditional solid fuel stoves.
3. Maximize exclusive LPG use and minimize use of solid fuels among intervention households.
4. Monitor solid fuel stove use and reinforce exclusive LPG use in intervention households that continue to use solid fuels for cooking
5. Avoid emphasizing potential health benefits of LPG to minimize the risk of introducing bias when participants report health outcomes.

Formative Research

Theoretical grounding

We used the COM-B and BCW to guide the design of formative research activities and to apply findings to the development of behavioral interventions. The COM-B Model is a behavioral system that provides a foundation for evaluating the capabilities, opportunities, and motivations that drive behavior, highlighting that a "behavioral diagnosis" must be understood to develop effective interventions.[37] The components of the COM-B map onto the theoretically-derived determinants of behavior from the TDF.[35] The TDF is comprised of 14 theoretical domain functions (Knowledge; Skills; Social/Professional Role and Identity; Beliefs about Capabilities; Optimism; Beliefs about Consequences; Reinforcement; Intentions; Goals, Memory, Attention and Decision Processes; Environmental Context and Resources; Social Influences; Emotions; and Behavioral Regulation) synthesized from 33 theoretical models and 128 constructs derived

For peer review only

For peer review only - http://bmjopen.bmj.com/site/about/guidelines.xhtml

from these models.[35,45] The BCW includes nine intervention functions (education, persuasion, incentivization, coercion, training, enablement, modeling, environmental restructuring, and restrictions) that can be applied to address gaps in identified capabilities, opportunities, and motivations to promote behavior change.[36,37] Using the COM-B, TDF, and BCW frameworks, we selected relevant domains and functions to develop behavioral strategies that are contextually specific (across HAPIN research sites) and grounded in theory.

Study Sites

Formative research was conducted in rural communities of Jalapa Department, Guatemala; Puno Province, Peru; Kayonza District, Eastern Province, Rwanda; and Nagapattinam and Kallakurichi (previously Villupuram) Districts in Tamil Nadu, India. Households in these rural communities were located between 30 minutes and up to several hours from main cities and varied in population density (Jalapa Department density 170/km^2; Kayonza District density 180/km^2; Puno Province density 18/km^2; Nagapattinam District density 615/km^2 and Kallakurichi District density 480/km^2). Formative research surveys conducted in the communities found LPG stove ownership to be 0% in Rwanda, 68% in Peru[46], 31% in Guatemala, and 57% in India. However, exclusive use of LPG stoves was lower: 0% in Rwanda, 3.5% in Peru[46], and 7% in Guatemala. In India, only 29.5% used primarily LPG (data on exclusive LPG use was not available).

In depth interviews, rapid assessments, and focus group discussions

In-depth interviews (IDIs) were conducted using semi-structured interview guides, tailored for each research site (Table 1). Participants were selected based on the following criteria: living in a

For peer review only - http://bmjopen.bmj.com/site/about/guidelines.xhtml

rural community in the country-specific study site, female, between the ages of 18-68, and able to understand and provide consent. In each site, we aimed to include participants with and without previous knowledge and/or use of LPG. Teams in India and Guatemala also sought to include some men meeting the same criteria. The following themes were covered in IDIs:

1. Stoves owned/frequency of use
2. Preferred stoves for traditional dishes/beverages
3. Family influences on stove/fuel use
4. Temporal, seasonal, and circumstantial influences on stove choice
5. Perceived benefits/disadvantages of traditional stoves
6. Knowledge/perceptions of LPG stoves
7. Reasons for stove stacking
8. Fuel purchase/solid fuel collection practices
9. Perceived impact of LPG on daily life/household status
10. Cooking tasks/consumption patterns, including during pregnancy/after birth

In Rwanda, cooking demonstrations and food tasting tests were conducted prior to IDIs in participating homes who lacked exposure to LPG. Several focus group discussions (FGDs) were conducted in Rwanda to develop materials given minimal familiarity with LPG in the study area.

Behavior change materials were developed based on IDI and FGD findings by local teams. FGDs were then conducted with participants in Rwanda, India, and Peru according to the same eligibility criteria as IDIs to review draft materials (Table 1). Participants were asked to describe their understanding of the messages being conveyed, any barriers and facilitators to LPG use not

For peer review only - http://bmjopen.bmj.com/site/about/guidelines.xhtml

captured, whether messages could be understood based solely on the pictures (given low literacy rates), and whether participants felt represented by the images. Given the extensive governmental support and ubiquity of LPG in India, FGDs aimed to identify a minimum set of information necessary for promoting exclusive LPG use among intervention households to minimize contamination bias in control households. Materials were modified based on FGD feedback.

Pilot studies with LPG cooking equipment

Following development of the behavior change materials, we conducted pilot studies to test and revise procedures for the main trial, test the effectiveness and acceptability of the behavioral strategies, and estimate anticipated $PM_{2.5}$ and black carbon levels (HAP results will be published separately).[47] Eligibility criteria included: female, primary cook, 18-34 years of age, living in a rural community in the country-specific study site, pregnant (<20 weeks gestation), non-smoker, and reliance on biomass fuel for cooking. Women in India (n=40), Rwanda (n=40), and Guatemala (n=60) were provided LPG stoves, free fuel for two months, and behavior change messages. We tested the effectiveness of the behavioral messages by assessing the rate of exclusive LPG use monitored by temperature data loggers (Dots™)[48,49] on stoves and acceptability through feedback from participants and field staff. Teams in Guatemala and Rwanda conducted FGDs with pilot household participants to revise behavior change materials. In Peru, behavioral messages were developed and tested with non-pregnant adult women in the Cardiopulmonary outcomes and Household Air Pollution (CHAP) trial[13]; messages specific to pregnant women and new mothers were assessed through interviews and FGDs with pregnant women or mothers with children under 2 selected according to the criteria explained above.

For peer review only - http://bmjopen.bmj.com/site/about/guidelines.xhtml

Behavioral strategy development

After finalizing the behavioral messages, a questionnaire and instruction sheet were developed using the COM-B model and BCW. These will guide the implementation of messages as part of a larger behavior change strategy and will be used to monitor the effectiveness in achieving exclusive LPG use in the HAPIN trial.

Ethics Approval

The formative research protocol was reviewed and approved by the Institutional Review Boards of Emory University (00089799); the Bloomberg School of Public Health, Johns Hopkins University (00007464); Asociación Benéfica PRISMA in Peru (CE3571.16); Sri Ramachandra Institute of Higher Education and Research (IEC-N1/16/JUL/54/49); the Indian Council of Medical Research–Health Ministry Screening Committee (5/8/4-30/(Env)/Indo-US/2016-NCD-I); Universidad del Valle de Guatemala (146-08-2016); the Guatemalan Ministry of Health National Ethics Committee (11-2016); the London School of Hygiene and Tropical Medicine (11664-2); and the Rwandan National Ethics Committee (No.148/RNEC/2017). The HAPIN trial is registered with clinicaltrials.gov (NCT02944682).

Patient and public involvement

The formative research reported in this manuscript was explicitly designed to engage community members at all four research sites in the design of an LPG intervention and behavioral reinforcement package to be implemented in the main HAPIN trial. Community members were involved in the initial identification of messages for promoting exclusive LPG use, as well as the refinement of the materials and methods for delivering those messages.

For peer review only - http://bmjopen.bmj.com/site/about/guidelines.xhtml
For peer review only

Data analysis

Qualitative data from IDIs and FGDs were analyzed using thematic analysis, which is flexible and atheoretical and can be applied across a range of qualitative methodologies.[50] Thematic analysis assists in the identification and organization of patterns in the data.[50] We used both an inductive and deductive approach. In Guatemala, data were transcribed and coded using HyperRESEARCH Software (Randolph, MA). Other country sites used Microsoft Excel to track themes and relevant quotes. Each country site analyzed their own data, which the first authors compiled for this manuscript.

RESULTS

Table 1 summarizes the formative research activities conducted in each research site.

Table 1. Formative research methods to design a behavioral intervention for the HAPIN trial

	Guatemala	India	Peru	Rwanda
Participant observations<br><br>Cooking demonstra-tions	Participant observations of cooking activities in 36 homes with LPG and wood stoves, 2-3 hours in each home	N/A*	N/A*	18 two-hour LPG cooking demonstrations and blind food tasting with non-LPG users (participants did not keep LPG stoves or cylinders)
In-Depth Interviews	18 interviews with women (primary cooks; 26-68 years of age) and 6 group interviews with 3 or 4 male participants	25 interviews, 11 in Nagapattinam and 14 in Kallakurichi (previously Villupuram) (23 female cooks, 2 men; 6 solid fuel users, 4 LPG users, 15 mixed fuel users; 21-65 years of age)	7 interviews (6 pregnant women, 1 new mother)	54 interviews with female primary cooks (14 LPG users, 22 non-LPG users, and repeat interviews with 18 of the same non-LPG users after an LPG cooking demonstration)

For peer review only - http://bmjopen.bmj.com/site/about/guidelines.xhtml

Key Informant Discussions	1 informal interview with an LPG distributor in Jalapa and 1 informal interview with a stove manufacturer	Informal discussions with LPG distributors and managers, and local community members	Informal discussions with field staff native to Puno	12 informal interviews with local field staff who installed the LPG stove and delivered behavioral training during the pilot study
Focus Group Discussions (FGDs)	9 FGDs of 5-6 participants (51 women; 2 men)	Two informal social group discussions (one in each site) with local villagers	1 FGD, 7 participants (4 pregnant women, 3 new mothers)	5 FGDs to develop behavior change materials (4 participants per group; 18-68 years of age), 4 FGDs to refine materials with pilot participants (2 FGDs after 1-month of LPG use, 2 FGDs after 2-months of LPG use; women 18-33 years of age; 0-2 children per household; 3-7 participants per group)
LPG stove pilot study	Behavioral messages reviewed upon LPG stove installation and reinforced at LPG cylinder delivery visits in 60 households over a 3 month period	Behavioral messages delivered at LPG stove installation to the 20 pilot intervention households	N/A (messages and materials piloted through CHAP study)[13]	Behavioral messages and materials delivered to 40 pilot study households

* Participant observations and cooking demonstrations were not conducted in Peru or India given widespread awareness of LPG and previous research in these areas.[32]

For peer review only

Formative research results

We identified nine main themes that influence exclusive LPG use: 1) Perceived disadvantages of solid fuel stoves, 2) Family influences on cooking decisions, 3) Traditional cookware and stoves on which they are used, 4) Traditional foods and preferences for stoves used to prepare them, 5) Other non-food related reasons for cooking, 6) Heating needs, 7) Previous awareness and

For peer review only - http://bmjopen.bmj.com/site/about/guidelines.xhtml

experience with LPG, 8) Safety concerns, and 9) Cost and availability of LPG. We provide a brief description of the themes below; specific sub-themes are summarized in Table 2.

Table 2. Summary of qualitative findings according to identified themes across study sites.

	Guatemala	India	Peru	Rwanda
1. Perceived disadvantages of solid fuel stoves				
Smoke is physically irritating	X	X	X	X
Solid fuel stoves dirty kitchens, cookware, clothes, and hands	X	X	X	X
Collecting and cooking with solid fuels requires time and energy costs	X	X	X	X
Monetary costs of solid fuel	X			X
Fear of snakes and environmental hazards when collecting fuel	X	X	X	X
Difficulty collecting and lighting wet solid fuel		X	X	
2. Family influences on cooking practices				
Family complaints that food gets cold quickly with LPG	X		X	
Family complaints that food cooked with LPG lacks flavor			X	
Family preference for food cooked with LPG because food does not taste like smoke				X
Family preference for LPG because food cooks faster	X	X	X	X
Family perception that LPG represents modernity		X		X
Husbands believe smoke harms their wives, but not husbands who do not cook	X			
3. Cookware				
Belief that commonly used clay pots cannot be used on LPG stoves	X		X	
Large, flat griddle required for tortillas	X			
Large pots required to cook staple foods	X			X
Meat, fish, and vegetables commonly roasted on open fires				X
4. Traditional food				
Perception that some traditional dishes taste better when cooked with solid fuel	X		X	X
Preference to cook food with solid fuel for family festivities and special occasions		X	X	X
Preference to cook beans with solid fuel	X			X
5. Other stove uses				
Heating water for bathing and washing	X	X	X	X

For peer review only - http://bmjopen.bmj.com/site/about/guidelines.xhtml

Cooking food for animals			X	
Making alcoholic beverages				X
6. Home heating needs				
Warmth from traditional stove viewed as beneficial during cold months	X		X	
7. LPG awareness				
Active governmental LPG campaigns have achieved high LPG awareness		X	X	
Low LPG awareness in countries that lack governmental LPG campaigns	X			X
8. LPG fears and safety				
Fear of LPG leaks and explosions or fires	X		X	X
Fear of improperly attaching regulator and hose to the LPG cylinder			X	X
Fear of LPG-related burns			X	X
Concerns for child safety	X		X	X
Mistrust of LPG providers	X		X	
9. LPG cost, supply, and distribution				
LPG refills perceived as expensive	X	X	X	X
Large and highly-regulated governmental LPG market		X		
Fewer governmental controls on LPG market	X		X	X
Lack of LPG sale points and delivery capability in study areas	X		X	X
Households are difficult to access (large distances between homes, lack of roads for transport)	X		X	

Reasons for abandonment of solid fuel stoves

Participants identified several disadvantages of solid fuel stoves, which suggest potential reasons for abandonment of traditional stoves.

Family preferences for cooking practices

Many participants mentioned that family preferences influenced decisions about which stove to use for cooking tasks.

For peer review only - http://bmjopen.bmj.com/site/about/guidelines.xhtml

Cookware

In Guatemala, Peru, and Rwanda, participants raised concerns that LPG stoves would not accommodate the pots and cookware they needed to cook local staple foods.

Traditional food

All sites, except India, identified traditional foods that people preferred to prepare with solid fuel stoves. Participants in Peru reported preferring to make a steamed quinoa bread (*quispiño*) with the traditional stove. In Guatemala and Rwanda, participants preferred to cook beans on the open fire because they believed beans cooked more slowly on LPG stoves. Additionally, in Rwanda, *ugali*, or cassava bread, is difficult to make on an LPG stove because of the force required to stir the dough, which could cause the burner grate to break.

Other uses of the stove

Traditional stoves are often used for purposes other than family meals, such as heating water for bathing during cold months. In Peru, people also commonly cook food for pigs and dogs. In Rwanda, open fires are sometimes used to make sorghum beer in large pots.

Home heating needs

Warmth emanating from the traditional stove was valued during cold months in Guatemala and Peru, and to a lesser extent in India. In Guatemala, participants used the traditional stove for space heating but said they would forgo this if they had free LPG. In Peru, participants described using extra layers of clothing instead of lighting their traditional stove for heat.

For peer review only - http://bmjopen.bmj.com/site/about/guidelines.xhtml

LPG awareness

In Peru and India, where governmental campaigns are actively promoting LPG nationwide, LPG awareness was much higher than in Guatemala and Rwanda, where no national LPG campaigns currently exist. Owning an LPG stove in India was considered highly aspirational.

LPG fears and safety

Participants reported some fears and concerns about LPG stove and cylinder safety, such as leaks, explosions, burns, and child safety. Several participants in Peru and Guatemala reported a lack of trust in the safety and reliability of products from some LPG companies. In India, participants' concerns about the safety of LPG were described as minimal and acceptable in light of other LPG benefits.

LPG cost, supply, and distribution

LPG refill costs were major barriers in all sites. Distance and inaccessibility of households also limited LPG cylinder refills. While the LPG market in India is extensive and highly regulated, there are fewer governmental controls and LPG sale points in Guatemala, Rwanda, and Peru.

Developing behavioral messages for the HAPIN trial

We mapped formative research findings onto the COM-B and TDF domains and developed behavioral messages to address identified themes and domains (Table 3). Factors related to capabilities and skills will be addressed using how-to materials and training, whereas factors related to motivation will be targeted with appeals to emotions such as trust, security, and conscious decision-making. Factors related to opportunity and context address physical

For peer review only

opportunities (providing prompt gas delivery and stove repair) as well as social opportunities (educating other members in the home to use the LPG stove) will be integrated into trial procedures.

Using the BCW, we identified seven intervention functions we will use to deliver messages that might lead to exclusive LPG use: *education* to increase knowledge and confidence in safe LPG use, *persuasion* to promote positive feelings about LPG benefits, *training* to enable LPG use to meet household needs, *environmental restructuring* to situate the LPG stove in kitchens that are free of smoke, *modeling* LPG stove use through hands-on training such as demonstrations of stove operation, *incentivization* by providing free LPG gas, and *enablement* by providing prompt LPG delivery and stove repairs. Because behavioral reinforcement visits are intended to be positive reinforcements and are not meant to be coercive or to induce negative emotions, two intervention functions, *restriction* and *coercion*, are not pertinent.

For peer review only

Table 3. Themes, behavioral messages, and strategies based on the Behavior Change Wheel developed during formative research for the HAPIN trial

Themes	Behavioral messages	COM-B/TDF domain[1]	Strategies and *related Intervention Functions*
1. Perceived disadvantages of solid fuel	Using gas prevents discomfort (by reducing smoke)<br>Gas can be used in all seasons/weather<br>Using gas is easy<br>Gas eliminates smoke in the home<br>Gas keeps hands, clothes, pots and kitchens cleaner<br>With gas you do not need to collect or buy solid fuel<br>Gas will not make holes in thatch/aluminum roof	*Motivation*/reinforcement; emotions; optimism; beliefs about consequences<br><br>*Opportunity*/environmental context and resources	• Emphasize disadvantages of traditional stoves to encourage abandonment of solid fuel<br>• *Education; Persuasion; Environmental restructuring*
2. Family influences	Tips for addressing concerns of household members<br>Tips for addressing concerns of friends/neighbors<br>You can keep foods hot, or reheat quickly, after cooking them with gas<br>Using gas saves money and time	*Motivation*/emotion; beliefs about capabilities; optimism<br><br>*Opportunity*/social influences	• Target behavioral interventions to all household members, not just primary cooks<br>• *Education; Persuasion; Enablement*
3. Cookware	Using clay and other pots on the gas stove<br>How to cook large quantities of food with gas<br>How to roast on an LPG stove	*Capability*/knowledge; skills	• Stove use demonstrations<br>• Guatemala and Rwanda: Provide cookware to enable typical cooking behaviors<br>• *Training; Modelling*
4. Traditional food	It is possible to cook beans on an LPG stove<br>How to cook traditional dishes with gas<br>How to enhance food flavor without solid fuel<br>How to make beer on an LPG stove<br>Practice makes perfect	*Capability*/knowledge; skills<br><br>*Motivation*/reinforcement; intentions; beliefs about capabilities; optimism	• Guatemala and Rwanda: Encourage soaking beans<br>• Rwanda: Emphasize removing large pots from stove for forceful stirring

For peer review only - http://bmjopen.bmj.com/site/about/guidelines.xhtml

			• *Education; Persuasion; Training*
5. Other stove uses	Everything can be done with gas	*Motivation*/goals; reinforcement; intentions; beliefs about capabilities<br><br>*Opportunity*/environmental context and resources	• Reassure households that LPG will be provided to meet all household cooking needs<br>• *Education; Persuasion; Incentivization; Environmental restructuring*
6. Home heating needs	How to stay warm when cooking with gas	*Motivation*/reinforcement; intentions; beliefs about consequences	• Emphasize that no stove should be used for heating home<br>• Emphasize other LPG benefits as trade-offs for lack of heat<br>• *Education; Persuasion*
7. LPG awareness	How to use LPG stove (turn off, turn on, open and close the gas)<br>How to regulate the flame, to prevent burning food and to save gas<br>How to clean stove	*Capability*/knowledge; skills; memory, attention and decision processes	• Hands-on training on stove operation<br>• *Education; Training*
8. LPG fears and safety	Gas is natural, like wood; the smell added to it is unpleasant to alert leaks, but not toxic<br>How to avoid burns<br>Child safety<br>If used correctly, LPG stoves are completely safe<br>How to check for and respond to a leak (soapy water)<br>How to change the cylinder<br>Explaining reasons why LPG brand can be trusted<br>Millions of people use LPG stoves with no problems<br>Who to call if there is a problem<br>Where/how to get technical support<br>How to store stove and gas cylinders properly	*Capability*/knowledge; skills; reinforcement; memory, attention and decision processes<br><br>*Motivation*/emotion; beliefs about capabilities	• Provide training on gas safety; provide phone numbers for project staff if leak detected or stove in need of repair; respond to household fears around gas use<br>• *Education; Persuasion; Training; Environmental restructuring; Enablement*
9. LPG costs,	Anticipating when gas will run out (cylinder check)	*Capability*/knowledge; skills; reinforcement; memory,	• Provide phone numbers for project

For peer review only - http://bmjopen.bmj.com/site/about/guidelines.xhtml

supply, and distribution	What to do when you need a gas refill (including when and who to call)<br>Security measures to prevent theft	attention and decision processes<br><br>*Opportunity*/environmental context and resources	staff if need gas refill; at installation instruct on secure storage of stove and cylinders<br>• *Enablement; Education; Persuasion; Training; Environmental restructuring*

[1]Examples of theoretical domains are provided, but are not exhaustive

For peer review only

Protocol for delivering behavioral strategies during the HAPIN trial

Stove package and equipment

Free, unlimited supply of LPG will be provided to intervention arm participants in the HAPIN trial to incentivize exclusive LPG use. To ensure constant supply (intervention function: environmental restructuring), two LPG cylinders will be provided. In Guatemala and Rwanda, the cylinders will have T-valve regulators with a flow switch that can be toggled to a second full tank when the first is empty. In India and Peru, families will be instructed to manually move the regulator between the cylinders. In Guatemala, the two cylinders will be installed outside the kitchen with a protective barrier. In Rwanda and Peru, cylinders will be installed inside, due to potential theft and freezing temperatures, respectively. Guatemala, Peru, and Rwanda will provide a three-burner stove and India will provide a two-burner stove, deemed to fulfill cooking needs during formative research. In Rwanda and India, tables will be provided for the LPG stove; in Peru and Guatemala, table-height stoves will be provided. To assure that traditional foods will be cooked on the LPG stove, the Guatemalan stove will include a griddle (*comal*) for cooking tortillas and households will receive a set of enamel pots. In Peru, households will be instructed to grease clay pots before using on the gas stove to prevent cracking. Households in Rwanda will be given a roasting appliance for grilling meats and vegetables.

Stove use pledge

When the LPG equipment is installed in intervention households in the HAPIN trial, field staff will ask all household members to be present and will administer a verbal pledge. By completing the pledge, participants will affirm that they; a) understand the study goals of reducing smoke exposures and achieving exclusive LPG use, b) that any type of food can be cooked with LPG, c)

For peer review only - http://bmjopen.bmj.com/site/about/guidelines.xhtml

that the LPG stove should be used only for household cooking needs, d) that the stove/cylinder should not be sold or rented, e) that HAPIN staff are available to help with any challenges related to the LPG stove, and f) that all household members intend to use the LPG stove exclusively (intervention function: persuasion).

Stove installation and training

At the LPG stove installation visit in the HAPIN trial, trained field technicians will provide training on: 1) lighting/adjusting the gas flame, 2) cleaning/maintaining the stove, 3) detecting/responding to gas leaks, 4) requesting cylinder refills and stove/cylinder repairs, 5) safe handling/use of cylinders and regulators, 6) benefits of LPG, and 7) disadvantages of solid fuel. In India, authorized technicians will collaborate with HAPIN staff to provide this training. In Rwanda, households will be required to pass a certification exam, demonstrating their ability to correctly perform the steps for safe stove and cylinder use before LPG stove installation (intervention functions: education and training)

Printed materials (calendars, booklets, pamphlets, posters)

At stove installation, study staff in Guatemala, Peru, and Rwanda will use a flipchart to deliver behavioral messages to participants. Participants in Guatemala, Rwanda, and Peru will also receive a printed guide, calendar, and/or poster containing pictorial and written representations of the behavioral messages to keep in their homes. In India, a flyer showing that a range of potential cooking tasks should be performed with LPG instead of the traditional stove will be left with households. Because LPG is highly aspirational and increasingly available through governmental

For peer review only

programs in India, printed materials on LPG benefits and traditional stove disadvantages will not be given to households to minimize unintended dissemination to control households.

Videos

In Guatemala and Rwanda, videos on safe stove and cylinder use, how to check for and respond to a gas leak, cleaning the gas stove, and cooking beans and other local dishes will be shown on a tablet to participants. Videos prepared in Rwanda will feature testimonials from both male and female LPG users, given formative research findings that men have a large influence over household decision-making.

Monitoring and reinforcing LPG use during the HAPIN trial

The following sections outline how we will monitor behavioral strategy effectiveness to achieve exclusive LPG use and how we will identify households that continue to use solid fuel for behavioral reinforcement visits in the HAPIN trial.

Stove use monitoring

Temperature data loggers known as Dots^{TM} (Geocene, Vallejo, CA, USA) will be installed on all solid fuel stoves in intervention households.[48] The Dots^{TM} data loggers will be placed near or within the combustion zone to provide clear temperature signals at five-minute sampling intervals. Using a mobile application, field teams will download data from each Dot^{TM} every two weeks to be analyzed on a secure cloud-based server. A deterministic algorithm will be used to identify rapid, sustained temperature increases, which will be flagged as traditional stove use events (Figure 1). Every week, local field staff, with periodic oversight by the BEC, will review

For peer review only - http://bmjopen.bmj.com/site/about/guidelines.xhtml

households with flagged traditional stove use events based on the Dot™ data. They will use this data to schedule reinforcement visits, as described below.

Observations of traditional stove use

Intervention households may build new makeshift fires that are not monitored by a temperature sensor or may remove the sensors from monitored stoves if they want to cook with solid fuel. Therefore, we will incorporate direct observations of traditional stove use into study activities. Field staff will conduct these observations at least once and up to three times per month in all intervention households. Using a checklist, staff will look for signs of recent traditional stove use, such as use during the visit, fresh ashes, hot embers, stoves that are warm to the touch, fresh blackening on walls, or lingering smoke.

Data tracking

Monthly LPG use will be monitored by LPG delivery staff, based on the frequency of refills provided to households. In households using less LPG than average, staff will assess whether supplemental solid fuel stove use is occurring. In households with high LPG usage, staff will confirm that the LPG stove is being used properly, i.e. not shared with neighbors, not used to prepare food for sale, appropriate flame settings to avoid fuel waste, and lids on pots. In Guatemala, Rwanda, and Peru, HAPIN staff will deliver LPG cylinder refills. In India, local LPG companies will deliver LPG with oversight from HAPIN staff.

LPG use reinforcement visits

Field staff will review the Dot™ data, observations, and LPG data described above to identify HAPIN trial households using solid fuel stoves. Within one week of identifying the household,

For peer review only - http://bmjopen.bmj.com/site/about/guidelines.xhtml

field staff will visit to reinforce abandonment of the solid fuel stove and promote exclusive LPG use. A questionnaire will be administered to elicit concerns or challenges related to the LPG stove, allowing field staff to address their specific problems. For example, if participants mention that they cannot cook traditional dishes on the LPG stove, the field technician may show a how-to video or explain how to cook that dish using LPG. If participants are anxious about switching the valve between LPG cylinders, the staff will demonstrate the process and coach the participant to perform the procedure. In Guatemala, behavioral staff will observe cooking and conduct demonstrations with intervention participants who use their traditional stove. In Rwanda, LPG testimonial videos featuring local families will be used to demonstrate benefits of LPG use.

Questionnaire on perceptions of LPG

A questionnaire on household perceptions of the LPG stove will be administered twice during pregnancy and twice after childbirth with HAPIN intervention households. The questionnaire, based on the COM-B and BCW will uncover additional barriers and facilitators related to LPG use that could be incorporated into behavioral messaging as the main trial progresses.

DISCUSSION

We identified nine potential influencers of exclusive LPG use at the household level, including perceived disadvantages of solid fuel, family preferences, cookware, traditional foods, non-food related cooking, heating needs, LPG awareness, safety, and cost and availability of fuel. These factors are similar to those found by Puzzolo et al. (2016) in their systematic review of cleaner fuel use.[20] Our study is unique because we used formative research grounded in behavior change

For peer review only - http://bmjopen.bmj.com/site/about/guidelines.xhtml

theory to design behavioral strategies to promote exclusive use of LPG in intervention households and solid fuel stove abandonment for the HAPIN trial.

Too often, interventions assume that introduction of cleaner fuels and technologies alone will be enough to eliminate HAP exposure. However, without a clear understanding and targeted approach to address cooking behaviors, family dynamics, and environmental constraints, households often resume use of solid fuel stoves for some or most of their cooking needs.[14] Our research was guided by an overarching set of common principles generalizable across contexts, but also uncovered contextual differences requiring tailored behavioral approaches. All behavioral strategies are intended to increase LPG adoption among intervention households with some contextualization to local conditions (e.g. climate differences, cooking practices) during the HAPIN trial.

Achieving exclusive use of new cooking technologies requires that study participants abandon, or de-implement, the old cooking technology. Such de-implementation has been used in behavioral intervention studies to change low-value practices or harmful behaviors.[44] Everett Rogers' diffusion of innovation theory suggests that households are more likely to abandon an old technology in favor of a new one when the new device has relative advantages over the old one, is compatible with local practices, and is not too complex to use.[51] During our formative phase, we provided 40-60 homes in Guatemala, India, and Rwanda with LPG stoves for two months to test acceptability, appropriateness, and feasibility of LPG stove and fuel use.[47] This initial phase enabled us to identify local perceptions of the relative advantages of LPG over traditional stoves, local practices that needed to be framed as compatible with LPG, and how to reduce complexity

For peer review only - http://bmjopen.bmj.com/site/about/guidelines.xhtml

of the LPG technology that we incorporated into training and behavior change strategies in each research site.[51]

Our formative research highlighted several areas that build upon efforts of previous cookstove trials. For example, self-reported stove use has been shown to over-estimate use of the improved or cleaner stove and under-estimate continued use of the solid fuel stove.[52] Other studies have used temperature data loggers to monitor the cleaner stove but did not monitor all solid fuel stoves in the home, which limits the ability to estimate stove stacking.[53] To better understand stove use and stacking behaviors, our study applies temperature data loggers on all traditional stoves with observations of traditional stove use at monthly home visits. Real time data summaries will allow continuous follow-up during the trial, flagging households using traditional stoves. Field staff will visit homes to troubleshoot potential LPG stove problems or other barriers and reinforce exclusive LPG use. Observations and responses to questionnaires on LPG perceptions and use will inform continuous adaptation to behavioral messages to maximize LPG adoption.

We designed our behavioral messaging to emphasize immediately visible disadvantages of cooking with solid fuels such as dirty kitchens and physical discomfort to encourage abandonment, based on our formative research that suggested these disadvantages were more tangible than long-term health effects. Other studies have also found focusing on health risks to be less effective.[32] Addressing context-specific fears and concerns, grounded in theory, may prove to be more effective than solely addressing capabilities, or skills training, on how to use the LPG stove. While skills training is essential for adoption of unfamiliar technologies,

For peer review only - http://bmjopen.bmj.com/site/about/guidelines.xhtml

additional behavior change messages that target motivations and opportunities among all household members may encourage a more complete household transition to exclusive LPG use. The TDF describes motivations, or social norms, as an essential part of designing behavioral interventions, and household members may either support or thwart the use of a new stove technology. Because the trial will provide free LPG, we will target opportunities by addressing environmental resources and context. This will assure that participants will be able to use the LPG stove for all purposes, including cooking animal fodder and brewing beer, which is uncommon when people pay for their own fuel.[20]

Cost remains one of the main drivers of cleaner fuel adoption.[20,22,23] Both monetary and time costs of obtaining cleaner fuel are frequent barriers to adoption.[23,46] In many rural areas, LPG cylinders are not delivered to homes, requiring families to travel long distances to procure fuel.[54] The HAPIN trial will provide 18 months of free fuel delivered to intervention households to overcome economic and transportation barriers and promote exclusive LPG use. Our formative research highlighted additional factors unrelated to cost that we hypothesize must also be addressed to achieve exclusive LPG use, such as reinforcing perceived disadvantages of cooking with solid fuel, addressing fears of LPG, fulfilling non-cooking needs for stove use such as heating and preparing animal fodder, and ensuring that LPG cooking is compatible with traditional foods. An additional influencer of clean fuel adoption and sustained use is the powerful role of market forces that generate adequate supply and demand activities to meet the needs of households that wish to use cleaner fuels. Because the HAPIN trial will provide free fuel, we did not explore market forces during the formative research, but an aim of our future

For peer review only - http://bmjopen.bmj.com/site/about/guidelines.xhtml

work is to understand supply and demand for LPG in the HAPIN trial sites with the goal of facilitating post-trial access to clean fuels.

Several potential limitations should be noted. First, we may have missed important contextual factors during our formative research. For example, in multi-family households, one LPG stove per household may not be sufficient to meet everyone's needs. Additionally, positive behavioral reinforcements may not be sufficient for intervention households that refuse to abandon solid fuel stoves. The complexity of changing cooking behaviors is one of the greatest challenges in stove adoption studies.[29,55,56] Second, our monitoring strategies may not accurately flag traditional stove use, which may result in unnecessary behavioral reinforcement visits to compliant households. Third, while we will track monthly LPG usage to assure that LPG households are requesting refills, LPG usage varies based on differences in household cooking tasks, family size, and other factors. Thus, we may incorrectly flag low LPG users for reinforcement. However, our extensive monitoring of stove use through observations, stove use questionnaires, and Dot™ data loggers will allow triangulation and offer insights into reasons for use and non-use of the LPG intervention over the 18-month trial. Lastly, our formative research, behavior change intervention, and monitoring plans are extensive and may not be feasible in all contexts. The HAPIN trial is not designed to determine which aspects of the intervention are critical for achieving exclusive LPG use, but rather to do everything possible to achieve exclusive use. Future research will be needed to test which components, i.e. cost removal, home delivery, stove use training, behavioral reinforcement, etc., are necessary and sufficient to achieve exclusive LPG use.

For peer review only - http://bmjopen.bmj.com/site/about/guidelines.xhtml

CONCLUSION

Achieving the highest possible exclusive LPG use among intervention households is essential for understanding the potential exposure reductions and health benefits that an LPG cooking intervention can provide. While our approach is more intensive than a real-world LPG promotion program, our formative research results provide valuable insights on how to develop, implement, monitor, and evaluate theory-informed behavioral strategies to promote LPG adoption and exclusive use. Strategies for promoting and monitoring exclusive LPG use are important not only to understand the impact of LPG adoption within trials, but also to sustain use in broader programs and promotional campaigns. While the behavioral components of the intervention were designed in the context of the HAPIN trial, the methods and lessons learned may provide insights for achieving sustained, exclusive use of cleaner fuels when delivered programmatically at scale.

For peer review only

For peer review only - http://bmjopen.bmj.com/site/about/guidelines.xhtml

FUNDING

The HAPIN trial is funded by the U.S. National Institutes of Health [cooperative agreement 1UM1HL134590] in collaboration with the Bill & Melinda Gates Foundation [OPP1131279]. The findings and conclusions in this report are those of the authors and do not necessarily represent the official position of the US National Institutes of Health, Department of Health and Human Services, or the Bill & Melinda Gates Foundation.

ACKNOWLEDGMENTS

A multidisciplinary, independent Data and Safety Monitoring Board (DSMB) appointed by the National Heart, Lung, and Blood Institute (NHLBI) monitors the quality of the data and protects the safety of patients enrolled in the HAPIN trial. NHLBI DSMB: Nancy R Cook, Stephen Hecht, Catherine Karr, Joseph Millum, Nalini Sathiakumar (Chair), Paul K Whelton, Gail G Weinmann (Executive Secretary). Program Coordination: Gail Rodgers, Bill & Melinda Gates Foundation; Claudia L Thompson, National Institute of Environmental Health Science; Mark J. Parascandola, National Cancer Institute; Danuta M Krotoski and Marion Koso-Thomas, Eunice Kennedy Shriver National Institute of Child Health and Human Development; Joshua P Rosenthal, Fogarty International Center; Conception R Nierras, NIH Office of Strategic Coordination Common Fund; Katie Kavounis, Dong- Yun Kim, Antonello Punturieri, and Barry S Schmetter, NHLBI.

Competing Interests:

The authors declare they have no actual or potential competing interests.

Contributors:

For peer review only

KNW and LMT led the writing of the manuscript. ZS and GR managed data collection and analysis in Rwanda. MH, ADA, and LMT managed data collection and analysis in Guatemala. GT and KB managed data collection and analysis in India. KNW, SAH, and WC managed data collection and analysis in Peru. AQ, EP, JP, TFC, JJM, and JPR provided overall guidance to study implementation. All authors contributed to the cross-site synthesis of findings, development of the study protocol, and writing and revision of the manuscript.

Data Sharing:

De-identified participant data are available upon reasonable request. Contact Kendra Williams (kendra.williams@jhu.edu) for Peru data, Lisa Thompson (lisa.thompson@emory.edu) for Guatemala data, Gurusamy Thangavel (thangavel@ehe.org.in) for India data, or Ghislaine Rosa (ghislaine.rosa@lshtm.ac.uk) for Rwanda data. For data re-use conditions, please contact Lance Waller (lwaller@emory.edu).

For peer review only

For peer review only - http://bmjopen.bmj.com/site/about/guidelines.xhtml

REFERENCES

1. World Health Organization. *Burning opportunity: Clean household energy for health, sustainable development, and wellbeing of women and children. (no. ISBN 978 92 4 156523 3, NLM classification: WA 754)*. Geneva, Switzerland. www.who.int; 2016.

2. Martin WJ, Glass RI, Araj H, et al. Household air pollution in low- and middle-income countries: Health risks and research priorities. *PLOS Medicine*. 2013;10(6):e1001455.

3. Bruce N, Pope D, Rehfuess E, Balakrishnan K, Adair-Rohani H, Dora C. WHO indoor air quality guidelines on household fuel combustion: Strategy implications of new evidence on interventions and exposure-risk functions. *Atmos Environ*. 2015;106:451-457.

4. Romieu I, Riojas-Rodriguez H, Marron-Mares AT, Schilmann A, Perez-Padilla R, Masera O. Improved biomass stove intervention in rural Mexico: Impact on the respiratory health of women. *Am J Respir Crit Care Med*. 2009;180(7):649-656.

5. Schilmann A, Riojas-Rodriguez H, Ramirez-Sedeno K, Berrueta VM, Perez-Padilla R, Romieu I. Children's respiratory health after an efficient biomass stove (patsari) intervention. *Ecohealth*. 2015;12(1):68-76.

6. Smith KR, McCracken JP, Weber MW, et al. Effect of reduction in household air pollution on childhood pneumonia in Guatemala (RESPIRE): A randomised controlled trial. *Lancet*. 2011;378(9804):1717-1726.

7. Hanna R, Duflo E, Greenstone M. Up in smoke: The influence of household behavior on the long-run impact of improved cooking stoves. *American Economic Journal: Economic Policy*. 2016;8(1):80-114.

For peer review only - http://bmjopen.bmj.com/site/about/guidelines.xhtml

8. Bensch G, Peters J. The intensive margin of technology adoption - experimental evidence on improved cooking stoves in rural Senegal. *J Health Econ*. 2015;42:44.

9. Mortimer K, Ndamala CB, Naunje AW, et al. A cleaner burning biomass-fuelled cookstove intervention to prevent pneumonia in children under 5 years old in rural Malawi (the cooking and pneumonia study): A cluster randomised controlled trial. *Lancet*. 2017;389(10065):167-175.

10. Alexander D, Northcross A, Wilson N, et al. Randomized controlled ethanol cookstove intervention and blood pressure in pregnant Nigerian women. *Am J Respir Crit Care Med*. 2017;195(12):1629-1639.

11. Alexander DA, Northcross A, Karrison T, et al. Pregnancy outcomes and ethanol cook stove intervention: A randomized-controlled trial in Ibadan, Nigeria. *Environ Int*. 2018;111:152-163.

12. Jack DW, Asante KP, Wylie BJ, et al. Ghana randomized air pollution and health study (GRAPHS): Study protocol for a randomized controlled trial. *Trials*. 2015;16:420-015-0930-8.

13. Fandino-Del-Rio M, Goodman D, Kephart JL, et al. Effects of a liquefied petroleum gas stove intervention on pollutant exposure and adult cardiopulmonary outcomes (CHAP): Study protocol for a randomized controlled trial. *Trials*. 2017;18(1):518.

14. Quinn AK, Bruce N, Puzzolo E, et al. An analysis of efforts to scale up clean household energy for cooking around the world. *Energy Sustain Dev*. 2018;46:1.

15. Pope D, Bruce N, Dherani M, Jagoe K, Rehfuess E. Real-life effectiveness of "improved" stoves and clean fuels in reducing $PM_{2.5}$ and CO: Systematic review and meta-analysis. *Environ Int*. 2017;101:7-18.

16. Johnson MA, Chiang RA. Quantitative guidance for stove usage and performance to achieve health and environmental targets. *Environ Health Perspect*. 2015;123(8):820-826.

For peer review only - http://bmjopen.bmj.com/site/about/guidelines.xhtml

17. World Health Organization (WHO). *WHO indoor air quality guidelines: Household fuel combustion.* www.who.int; 2014.

18. Goodwin NJ, O'Farrell SE, Jagoe K, et al. Use of behavior change techniques in clean cooking interventions: A review of the evidence and scorecard of effectiveness. *J Health Commun*. 2015;20(sup1):43-54.

19. Hanbury A, Farley K, Thompson C, Wilson PM, Chambers D, Holmes H. Immediate versus sustained effects: Interrupted time series analysis of a tailored intervention. *Implement Sci*. 2013;8:130-5908-8-130.

20. Puzzolo E, Pope D, Stanistreet D, Rehfuess EA, Bruce NG. Clean fuels for resource-poor settings: A systematic review of barriers and enablers to adoption and sustained use. *Environ Res*. 2016;146:218-234.

21. Smith KR. Changing paradigms in clean cooking. *EcoHealth*. 2015;12:196-199.

22. Abdulai MA, Afari-Asiedu S, Carrion D, et al. Experiences with the mass distribution of LPG stoves in rural communities of Ghana. *Ecohealth*. 2018;15(4):757-767.

23. Gould CF, Urpelainen J. LPG as a clean cooking fuel: Adoption, use, and impact in rural India. *Energ Policy*. 2018;122:395.

24. Ronzi S, Puzzolo E, Hyseni L, et al. Using photovoice methods as a community-based participatory research tool to advance uptake of clean cooking and improve health: The LPG adoption in Cameroon evaluation studies. *Soc Sci Med*. 2019;228:30-40.

25. Clean Cooking Alliance. Igniting change: A strategy for universal adoption of clean cookstoves and fuels. *Washington, DC*. 2011.

For peer review only

26. Evans WD, Johnson M, Jagoe K, et al. Evaluation of behavior change communication campaigns to promote modern cookstove purchase and use in lower middle income countries. *Int J Environ Res Public Health*. 2017;15(1):11. doi: 10.3390/ijerph15010011.

27. Dalaba M, Alirigia R, Mesenbring E, et al. Liquified petroleum gas (LPG) supply and demand for cooking in northern Ghana. *Ecohealth*. 2018;15(4):716-728.

28. Pope D, Bruce N, Higgerson J, et al. Household determinants of liquified petroleum gas (LPG) as a cooking fuel in south west Cameroon. *Ecohealth*. 2018;15(4):729-743.

29. Alam A, Tawale N, Patel A, Dibley MJ, Jadhao S, Raynes-Greenow C. Household air pollution intervention implications: Findings from qualitative studies and a field trial of clean cookstoves in two rural villages in India. *Int J Environ Res Public Health*. 2016;13(9):893. doi: 10.3390/ijerph13090893.

30. Rhodes EL, Dreibelbis R, Klasen EM, et al. Behavioral attitudes and preferences in cooking practices with traditional open-fire stoves in Peru, Nepal, and Kenya: Implications for improved cookstove interventions. *Int J Environ Res Public Health*. 2014;11(10):10310-10326.

31. Thompson LM, Hengstermann M, Weinstein JR, Diaz-Artiga A. Adoption of liquefied petroleum gas stoves in Guatemala: A mixed-methods study. *Ecohealth*. 2018;15(4):745-756.

32. Hollada J, Williams KN, Miele CH, Danz D, Harvey SA, Checkley W. Perceptions of improved biomass and liquefied petroleum gas stoves in Puno, Peru: Implications for promoting sustained and exclusive adoption of clean cooking technologies. *Int J Environ Res Public Health*. 2017;14(2):182.

33. Clean Cooking Alliance. Behavior change communication. https://www.cleancookingalliance.org/market-development/demand-creation/behavior-change-communication.html. Updated 2020. Accessed May 28, 2020.

For peer review only - http://bmjopen.bmj.com/site/about/guidelines.xhtml

34. Mosler HJ. A systematic approach to behavior change interventions for the water and sanitation sector in developing countries: A conceptual model, a review, and a guideline. *Int J Environ Health Res*. 2012;22(5):431-449.

35. Cane J, O'Connor D, Michie S. Validation of the theoretical domains framework for use in behaviour change and implementation research. *Implement Sci*. 2012;7:37-5908-7-37.

36. Michie S, Atkins L, West R. *The behaviour change wheel: A guide to designing interventions.* London: Silverback Publishing; 2014. www.behaviourchangewheel.com.

37. Michie S, van Stralen MM, West R. The behaviour change wheel: A new method for characterising and designing behaviour change interventions. *Implementation Science*. 2011;6(1):42.

38. Kwah KL, Fulton EA, Brown KE. Accessing national health service stop smoking services in the UK: A COM-B analysis of barriers and facilitators perceived by smokers, ex-smokers and stop smoking advisors. *Public Health*. 2019;171:123-130.

39. Gould GS, Bar-Zeev Y, Bovill M, et al. Designing an implementation intervention with the behaviour change wheel for health provider smoking cessation care for Australian indigenous pregnant women. *Implementation Science*. 2017;12(1):114.

40. Wodnik BK, Freeman MC, Ellis AS, Awino Ogutu E, Webb Girard A, Caruso BA. Development and application of novel caregiver hygiene behavior measures relating to food preparation, handwashing, and play environments in rural Kenya. *Int J Environ Res Public Health*. 2018;15(9):10.3390/ijerph15091994.

41. Carrion D, Dwommoh R, Tawiah T, et al. Enhancing LPG adoption in Ghana (ELAG): A factorial cluster-randomized controlled trial to enhance LPG adoption & sustained use. *BMC Public Health*. 2018;18(1):689-018-5622-3.

For peer review only - http://bmjopen.bmj.com/site/about/guidelines.xhtml

42. Clasen T, Checkley W, Peel JL, et al. Design and rationale of the HAPIN study: A multicountry randomized controlled trial to assess the effect of liquefied petroleum gas stove and continuous fuel distribution. *Environ Health Perspect*. 2020;128(4):047008.

43. Household air pollution and health: A multi-country LPG intervention trial (HAPIN). ClinicalTrials.gov identifier: NCT02944682. U.S. National Library of Medicine Web site. https://clinicaltrials.gov/ct2/show/NCT02944682. Published October 26, 2016. Updated May 6, 2019.

44. Thompson LM, Diaz-Artiga A, Weinstein JR, Handley MA. Designing a behavioral intervention using the COM-B model and the theoretical domains framework to promote gas stove use in rural Guatemala: A formative research study. *BMC Public Health*. 2018;18(1):253.

45. Michie S, Johnston M, Abraham C, et al. Making psychological theory useful for implementing evidence based practice: A consensus approach. *Qual Saf Health Care*. 2005;14(1):26-33.

46. Pollard SL, Williams KN, O'Brien CJ, et al. An evaluation of the Fondo de Inclusion Social Energetico program to promote access to liquefied petroleum gas in Peru. *Energy Sustain Dev*. 2018;46:82-93.

47. Liao J, McCracken JP, Piedrahita R, et al. The use of bluetooth low energy beacon systems to estimate indirect personal exposure to household air pollution. *J Expo Sci Environ Epidemiol*. 2019.

48. Geocene Inc. Geocene dots temperature loggers. *Vallejo, CA*. 2016;https://geocene.com;.

49. Wilson DL, Williams KN, Pillarisetti A. An integrated sensor data logging, survey, and analytics platform for field research and its application in HAPIN, a multi-center household energy intervention trial. *Sustainability*. 2020;12(5).

For peer review only - http://bmjopen.bmj.com/site/about/guidelines.xhtml

50. Braun V, Clarke V. Using thematic analysis in psychology. *Qualitative Research in Psychology*. 2006;3(2):77-101.

51. Rogers EM. *Diffusion of innovations.* 5th ed. New York: Free Press; 2003:551.

52. Lozier MJ, Sircar K, Christensen B, et al. Use of temperature sensors to determine exclusivity of improved stove use and associated household air pollution reductions in Kenya. *Environ Sci Technol*. 2016;50(8):4564-4571.

53. Ruiz-Mercado I, Lam N, Canuz E, Davila G, Smith KR. Low-cost temperature loggers as stove use monitors (SUMs). *Boiling Point*. 2008;55:16-18.

54. Asante KP, Afari-Asiedu S, Abdulai MA, et al. Ghana's rural liquefied petroleum gas program scale up: A case study. *Energy Sustain Dev*. 2018;46:94-102.

55. Mukhopadhyay R, Sambandam S, Pillarisetti A, et al. Cooking practices, air quality, and the acceptability of advanced cookstoves in Haryana, India: An exploratory study to inform large-scale interventions. *Glob Health Action*. 2012;5:1-13.

56. Hooper LG, Dieye Y, Ndiaye A, et al. Traditional cooking practices and preferences for stove features among women in rural Senegal: Informing improved cookstove design and interventions. *PLoS One*. 2018;13(11):e0206822.

For peer review only

FIGURE LEGEND

Figure 1. Geocene Dot^{TM} data from one household showing a flagged cooking event with a rapid temperature increase.

For peer review only

For peer review only - http://bmjopen.bmj.com/site/about/guidelines.xhtml

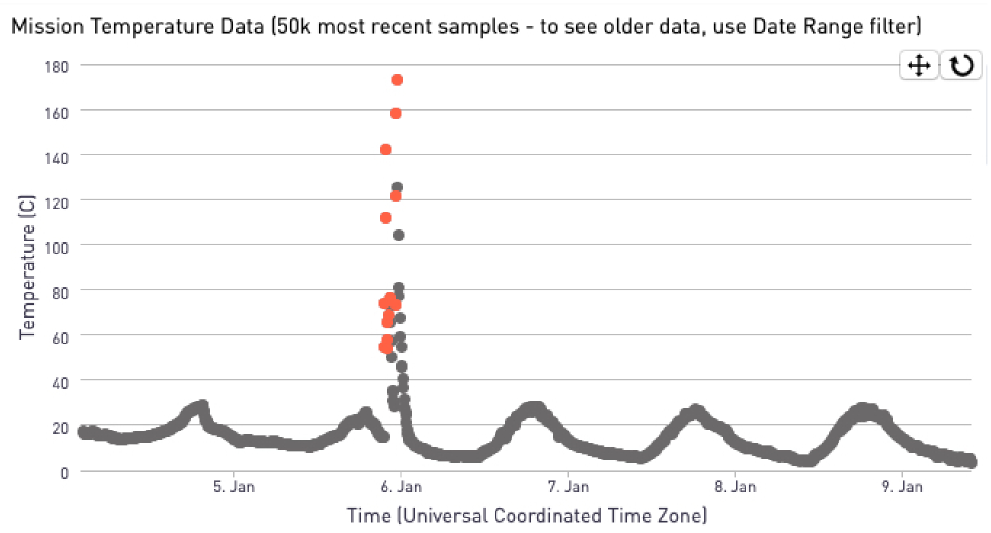

242x130mm (72 x 72 DPI)
